# Feeding ecology of the Terciopelo pit viper snake (*Bothrops asper*) in Ecuador

**DOI:** 10.7717/peerj.14817

**Published:** 2023-02-08

**Authors:** Amaru Loaiza-Lange, Diana Székely, Omar Torres-Carvajal, Nicolás Tinoco, David Salazar-Valenzuela, Paul Székely

**Affiliations:** 1 Museo de Zoología, Universidad Técnica Particular de Loja, Loja, Ecuador; 2 Departamento de Ciencias Biológicas y Agropecuarias, Laboratorio de Ecología Tropical y Servicios Ecosistémicos (EcoSs-Lab), Facultad de Ciencias Exactas y Naturales, Universidad Técnica Particular de Loja, Loja, Ecuador; 3 Research Center of the Department of Natural Sciences, Faculty of Natural and Agricultural Sciences, Ovidius University Constanţa, Constanţa, Romania; 4 Museo de Zoología, Escuela de Biología, Pontificia Universidad Católica del Ecuador, Quito, Ecuador; 5 Centro de Investigación de la Biodiversidad y Cambio Climático (BioCamb) e Ingeniería en Biodiversidad y Recursos Genéticos, Facultad de Ciencias del Medio Ambiente, Universidad Indoamérica, Quito, Ecuador

**Keywords:** Feeding habits, Viper, Ecology, Sexual dimorphism, Feeding strategies, Sit-and-wait

## Abstract

Thoroughly documenting prey items and diet composition is crucial for understanding a predator’s role in the ecosystem. In gape restricted predators, such as snakes, documenting and analyzing the type and size of the prey is important to interpret their ecological role. We describe the diet patterns of a species of venomous snake, the Terciopelo pit viper (*Bothrops asper*), from its Ecuadorian populations. Examining the gastrointestinal contents of museum specimens collected over an extensive area of the Pacific lowlands of Ecuador, we encountered 69 identifiable prey items from four major taxonomic groups (amphibians, centipedes, mammals, and reptiles). We evaluated the observed composition of prey to check for differences between sexes and size-classes. To complement our observations of the Terciopelo species complex throughout their distribution, we carried out a systematic literature review. Our data show an ontogenetic shift in diet, with a transition from more diverse diet in juveniles towards a mammal-specialized diet in adults, and distinct proportion of prey taxa between the sexes in the juvenile size class.

## Introduction

Predator-prey interactions play an important part in shaping ecosystems, with both predator and prey constantly adapting their functional roles (Gibert & DeLong, 2017; Schalk & Cove, 2018; Glaudas et al., 2019). Information on the relationship between a predator and the type and size of its prey can contribute to the understanding of the functioning of ecosystems (Reuman & Cohen, 2004; Schalk & Cove, 2018) and of the ecological impact that changes in prey availability can have on a population (Zipkin et al., 2020). This connection is well-known in snakes, given their unique feeding strategies (*e.g*., use of venom, constriction, luring, ambush, thermal detection, whole prey consumption; Greene, 1997), due to allometric constraints which had a major role in their feeding biology (Martins, Marques & Sazima, 2002). These strict relationships have the potential to radically change with sex or age (Shine, 1991).

The Terciopelo pit vipers, *Bothrops asper* (Garman, 1883) species complex (Salazar-Valenzuela, 2016; Saldarriaga-Córdoba et al., 2017; Salazar-Valenzuela et al., 2019; Mora-Obando et al., 2020), are highly adaptable venomous snakes distributed from eastern Mexico, throughout Central America, to Colombia and Venezuela (western and Caribbean), western lowlands of Ecuador, and down to the extreme northwestern Peru (Solórzano & Cerdas, 1989; Campbell & Lamar, 2004; Cisneros-Heredia & Touzet, 2004). They occupy a wide range of ecosystems and have successfully adapted to modified habitats (Campbell & Lamar, 2004; Sasa, Wasko & Lamar, 2009). Early dietary records indicate that the Terciopelo is a generalist predator, with a taxonomically diverse diet composed of prey items of various sizes (Mole, 1924; Parker, 1926; March, 1928; Barbour & Loveridge, 1929; Nicéforo-María, 1930; Smith, 1947). The most complete accounts of feeding patterns of *B. asper* come mainly from populations in Central America (Solórzano & Cerdas, 1989; Sasa, Wasko & Lamar, 2009; Wasko & Sasa, 2012). Sasa, Wasko & Lamar (2009) list 42 types of prey, with rodents being the most common (69%), followed by birds (10.3%), lizards (10.3%), and anurans (6.9%), and a significant difference between males and females in terms of the type of selected prey. Additionally, they found that adults consume larger prey masses relative to their body compared to juveniles and subadults. Kuch et al. (2004) and Boada et al. (2005) published the first accounts for populations in Ecuador, registering amphibians, birds, centipedes, insects, and mammals among prey items. Since these reports referred to specimens predominantly from the central-northern part of the country, a broader study that includes specimens from populations from the north and south of the country is needed, reflecting the lineage diversity shown by this species in Ecuador (Salazar-Valenzuela et al., 2019).

In this study, we analyze the diet of *B. asper* along the Pacific coast of Ecuador and provide a comprehensive list of prey items. We also test if niche and prey size are influenced by sexual maturity and/or sex. Additionally, we synthetize all available published data on the diet of the species complex available in the literature, generating a comprehensive list of prey types, countries, and publication type, providing a better understanding of the feeding habits of Terciopelo pit vipers.

## Materials and Methods

### Sampling and collections

To describe the diet of the *B. asper* complex in Ecuador (Salazar-Valenzuela et al., 2019), we examined the gastro-intestinal tract (GIT) contents of 193 specimens (105 females and 88 males) from the collections of Museo de Zoología de la Pontificia Universidad Católica del Ecuador (QCAZ) and Museo de Zoología, Universidad Técnica Particular de Loja (MUTPL). Most of the examined specimens were fixed in 10% formalin and stored in 75% ethanol. As some specimens were killed by humans, they showed considerable damage, especially near the head, preventing, in some cases, accurate morphological measurements.

Permits for this research (001-10 IC-FAU-DNB/MA, 005-12 IC-FAU-DNB/MA, 005-14 IC-87 FAU-DNB/MA, MAE-DNB-CM-2015-0016 and MAAE-ARSFC-2020-0727) were issued by Ministerio del Ambiente, Agua y Transición Ecológica del Ecuador.

### Predator-prey relationship

Before examination, each specimen was put in a slightly tilted tray for 30 min to drain as much of the liquid as possible, and, if necessary, dried with blotting paper. For each snake, body mass, snout-vent length (SVL), tail length, and diameter of the narrowest part of the neck were recorded. Measurements were performed with a caliper or a string to the nearest 1.0 mm (three consecutive measurements were performed for consistency and precision). Body mass was recorded with a precision scale to the nearest 0.1 g for specimens of less than 500 g, while larger specimens were measured to the nearest 1 g. Specimens were classified as juveniles (<60 cm), subadult males (60–98 cm), subadult females (60–110 cm), adult males (>98 cm) and adult females (>110 cm; Solórzano & Cerdas, 1989; Sasa, 2002; Boada et al., 2005). Sex was determined by tail dissection and/or observation of ovaries or testes during GIT inspection. A ventral incision was performed along the body to extract prey items from the snake’s digestive tract (stomach and small and large intestines); prey location (distance from the snake’s head) and direction of ingestion were recorded.

Subsequently, items found in the GIT were labelled and stored individually in flasks containing 75% ethanol. Depending on the state of digestion, prey items were classified as complete, incomplete, residuals (*e.g*., hairs, scales, and spines), or unidentifiable. Remains that could not be identified were excluded from further analysis. Each prey item was identified to the lowest taxonomic group possible, using specialized bibliography or with the aid of taxonomic experts. All identifiable items were later grouped into one of four main taxonomic prey categories: Amphibia (Anura), Chilopoda (Scolopendromorpha), Mammalia (Rodentia and Didelphimorphia), and Reptilia (Squamata). Following Martins, Marques & Sazima (2002) and Martins & Gordo (1993), insect remains found in the hindgut were inferred to be secondary ingestion resulting from an ingested amphibian. For each complete prey item, mass was recorded to the nearest 0.1 g, and both length (without tail) and diameter (largest width) were measured to the nearest 1.0 mm.

To study predator-prey relationships, we used the mass ratio (MR; prey mass/snake mass) (Greene, 1983; Pough & Groves, 1983; Martins, Marques & Sazima, 2002; Campbell & Lamar, 2004; Kuch et al., 2004; Boada et al., 2005; Sasa, Wasko & Lamar, 2009), in those cases where an accurate measurement of the prey was possible (*i.e*., complete, mostly undigested prey items). Additionally, we calculated the diameter ratio (DR; prey diameter/narrowest snake neck diameter), generally used in studies of sea snakes (Voris & Voris, 1983; Sanders et al., 2013). We chose this index instead of the ingestion ratio IR—prey width/snake head length (Martins, Marques & Sazima, 2002) since several specimens had unreliable head length measurements because their heads were damaged. We calculated the relative body mass (*i.e*., the body condition index or BCI), relative tail length and relative neck diameter as the residuals from the linear regression of log-transformed values of these measurements on log-transformed SVL, which allowed us to detect differences in shape while controlling for SVL (King, 1989; Sivan et al., 2020).

To assess the relative importance index of the main taxonomic categories, we adapted the formula proposed by Biavati, Wiederhecker & Colli (2004): *I*_x_ = (*n*_x_% + *f*_x_% + *m*_x_%)/3, where: *n*_x_% represents the percentage for each taxonomic group of prey from the total number of prey items; *f*_x_% represents the percentage of specimens that contained a particular type of prey from the total number of snakes that had prey or remains in their guts, and *m*_x_% represents the percentage of mass of the prey type from the total prey mass. We used the mass (*m*_x_) recorded for each complete prey item instead of the volume, since we consider it to be a more accurate measurement in our case, as it was shown in previous works (Feldman & Meiri, 2013). We calculated food niche breadth for each of the two sexes and for the three size-classes (juvenile, subadult, and adult) based on Simpson’s diversity index: $B = 1 - \sum {p_i}^2$, where *p*_i_ is the proportion of each type of prey (Simpson, 1949).

To evaluate if prey type differed between life stages or between sexes, we performed Chi-square tests. We checked if size-classes differed in the number of prey items with Kruskal–Wallis analysis. To determine the sexual dimorphism, we tested for differences in absolute values of SVL, mass and neck diameter, as well as in the BCI, relative tail length and neck diameter using Welch’s *t*-tests. To evaluate if the preference for the main taxonomic groups was influenced by snake sex or SVL, we fitted generalized linear models with a binomial distribution and logit link function (MASS package), using all snakes with identifiable GIT-contents (*n* = 46; one male was excluded due to unavailable SVL measurement). We used the full and null models for each predictor separately (Grainger et al., 2020); competing models were compared (package MuMIn) using the corrected Akaike information criterion (AIC_c_) and Akaike weight (w_*i*_), considering the model with the lowest AIC_c_ and highest w_*i*_ to be the best fit for the data (Burnham & Anderson, 2004).

To investigate the relationship between characteristics of the snake (SVL and sex) and the relative size of the prey they ingested, we used linear models with either MR or DR as the dependent variable and predator size and sex as predictors, applied over a filtered dataset (*i.e*., with incomplete prey items removed). All statistical analysis were performed using the R Statistical Software (R Core Team, 2021) with an *α* = 0.05.

### Bibliographic research

Various databases were searched for diet reports of *B. asper*: Scopus, Web of Science, Scielo, Google Scholar, and Biodiversity Heritage Library. As keywords, we used the scientific and common names and iterations given to this species throughout the years (*e.g*., “Fer-de-lance”, “*Bothrops*”, “*atrox*”, “*asper*”, “Terciopelo”, “barba amarilla”, “equis”), as well as the terms diet, feeding, food, single or in combination. We excluded reports of feeding in captivity.

## Results

### Predator-prey relationship

From the 193 analyzed specimens, we found GIT content in 47 individuals: 27 females and 20 males. Further analysis was carried out exclusively on specimens with GIT content. Five specimens belong to the Chocoan region lineage and 42 to the Pacific lowlands lineage (Salazar-Valenzuela et al., 2019; Mora-Obando et al., 2020), covering 12 Ecuadorian provinces (Table 1). This encompasses most of the species’ distribution in Ecuador, except for the populations from Río León and Vilcabamba (Southern Ecuador), since none contained prey items or remains in their gut. In terms of absolute size, the vipers showed a marked sexual dimorphism, females being both larger (SVL, *t*_40.47_ = 2.54, *p* = 0.015) and heavier (*t*_33.63_ = 2.68, *p* < 0.011) than males, as well as having wider necks (*t*_36.91_ = 3.5, *p* < 0.002). Females had a similar body condition to males (*t*_30.83_ = 0.75, *p* = 0.458). In terms of relative values, females had shorter tails (*t*_40.87_ = −3.56, *p* = 0.001) and wider necks (*t*_37.73_= 3.34, *p* < 0.002) when adjusted for SVL.

**Table 1:** General data of specimens of *Bothrops asper* used in this study from Ecuador, and their prey.

Snake information								Prey information			
Museum #	Province	Sex	Size-class	TL	SVL	ND	Mass	Taxa	L	D	M
MUTPL-45	El Oro	F	J	319	272	6.1	11	*Medopheos edracanthus*	95	11.7	8
								Squamata	n.a.	n.a	2
MUTPL-205	Guayas	F	S	789	685	10.3	139.9	Mammalia	n.a.	n.a	1.6
MUTPL-228	El Oro	F	A	1,148	983	14.3	515	Mammalia	n.a.	n.a	34.2
MUTPL-229	El Oro	F	A	1,246	1,061	19.2	556.8	Mammalia	n.a.	n.a	17.5
QCAZR-5053	Esmeraldas	F	J	381	338	6.76	14.7	*Leptodactylus ventrimaculatus*	111	16.27	6.6
								Cricetidae	n.a.	n.a	0.5
QCAZR-5657	S.D. de los Tsáchilas	F	J	n.a.	316	6.1	16.7	*Pristimantis* sp.	n.a.	n.a	0.2
								Anura	n.a.	n.a.	0.1
QCAZR-5760	Esmeraldas	M	S	726	632	7.63	53.38	Scolopendromorpha	117	13.18	7.72
								Scolopendromorpha	n.a.	n.a.	n.a.
QCAZR-5764	Esmeraldas	F	S	683	608	7.92	66.8	Cricetidae: *Oligoryzomys* sp.	146	25.9	26.3
QCAZR-5766	Esmeraldas	F	S	676	586	8.89	77.7	Cricetidae: *Transandinomys* sp.	17.1	29.1	35.6
								Cricetidae	n.a.	n.a.	1.1
								Scolopendromorpha	n.a.	n.a.	0.9
QCAZR-5768	Esmeraldas	M	S	722	616	9.22	95	Cricetidae: *Transandinomys* sp.	97	15.61	6.2
								Cricetidae: *Transandinomys* sp.	n.a.	n.a.	5.1
QCAZR-5850	Esmeraldas	M	J	360	310	6.09	17.1	Unidentifiable	n.a.	n.a.	n.a.
QCAZR-5856	Cotopaxi	M	J	464	400	6.08	28.6	Cricetidae: *Handleyomys* sp.	n.a.	n.a.	5.3
QCAZR-5858	Esmeraldas	F	A	1,485	1,314	19.15	915.3	Echimydae: *Proechimys semiespinosus*	425	n.a.	56.4
QCAZR-5862	Los Rios	F	S	941	821	12.61	235.5	Cricetidae	n.a.	n.a.	6.7
QCAZR-5871	Manabi	M	J	423	365	5.78	21.3	*Pristimantis* sp.	n.a.	n.a.	1.3
								Anura	n.a.	n.a.	0.2
QCAZR-5876	Esmeraldas	M	J	306	269	5.41	7.35	*Leptodactylus ventrimaculatus*	15.05	4.14	0.8
								Anura	13.34	3.83	0.8
								Anura	n.a.	n.a.	n.a.
QCAZR-5877	Esmeraldas	F	S	n.a.	847	10.31	226.45	Cricetidae: *Nephelomys moerex*	94	11.26	3.52
								Cricetidae	n.a.	n.a.	0.83
QCAZR-5879	Esmeraldas	F	J	502	441	10	27.03	Cricetidae: *Nephelomys moerex*	166	25.83	42.65
QCAZR-6435	Pichincha	M	S	684	585	9.66	83.6	Cricetidae	108.84	15.21	7.8
								Mammalia	n.a.	n.a.	3.5
QCAZR-6998	Esmeraldas	M	S	636	541	8.1	69.8	*Leptodactylus ventrimaculatus*	100	12.5	3.9
								Cricetidae: *Handleyomys*	n.a.	n.a.	n.a.
QCAZR-9126	El Oro	M	S	932	791	9.05	273.6	Cricetidae	n.a.	n.a.	7.6
QCAZR-9468	Loja	M	J	424	363	6.11	18.1	Scolopendromorpha	n.a.	n.a.	0.2
QCAZR-11284	Loja	F	S	734	641	11.36	115.9	Cricetidae: *Melanomys*	n.a.	n.a.	0.9
QCAZR-11613	Manabi	F	A	1,447	1,280	19.64	796	Cricetidae	145	n.a.	14.7
								Cricetidae	n.a.	n.a.	16.1
QCAZR-11614	Manabi	M	A	1,290	1,118	15.76	663	Cricetidae	n.a.	n.a.	8.3
QCAZR-11617	Manabi	M	S	786	676	8.03	71.1	Cricetidae: *Melanomys*	149	24.49	30.5
								Cricetidae	n.a.	n.a.	0.8
QCAZR-11627	Esmeraldas	F	A	1,455	1,280	24.76	1,414.5	Echimydae: *Proechimys* sp.	n.a.	n.a.	5.5
QCAZR-12449	El Oro	M	A	1,086	935	14.64	437	Cricetidae	n.a.	n.a.	4
QCAZR-12454	Guayas	M	J	n.a.	n.a.	4.53	9.3	unidentifiable	n.a.	n.a.	n.a.
QCAZR-12460	Santa Elena	F	A	1,304	1,145	19.18	854.5	Echimydae: *Proechimys semiespinosus*	n.a.	n.a.	18.5
QCAZR-12463	Manabi	F	J	557	491	7.5	37.6	*Anolis bitectus*	75	10.91	1.9
								Scolopendromorpha	58.52	12.89	2.5
								Scolopendromorpha	n.a.	n.a.	0.6
QCAZR-12497	Pichincha	M	J	484	416	6.28	27.9	unidentifiable	n.a.	n.a.	n.a.
QCAZR-12499	Pichincha	M	J	495	432	5.84	22.5	*Pristimantis achatinus*	99.78	15.55	5.2
QCAZR-12567	Loja	M	J	375	323	6.25	17.4	Anura	n.a.	n.a.	0.1
QCAZR-12570	Loja	F	A	1,555	1,378	21.52	1,099.8	Cricetidae	n.a.	n.a.	11.9
QCAZR-12574	Cotopaxi	M	S	787	682	9.1	115.1	Cricetidae: *Oligoryzomys* sp.	73.29	13.16	4.4
								Mammalia	n.a.	n.a.	6.2
QCAZR-12576	Cotopaxi	M	A	1,363	1,195	16.32	695.4	Cricetidae: *Oligoryzomys* sp.	n.a.	n.a.	5.5
								Cricetidae	n.a.	n.a.	22.1
QCAZR-12580	Cotopaxi	F	A	n.a.	1,264	24.32	1,251.99	Cricetidae	n.a.	n.a.	11.01
QCAZR-12581	Guayas	F	A	1,283	1,106	19.92	688.1	Cricetidae	n.a.	n.a.	1.9
QCAZR-12589	Manabi	M	S	619	528	8.08	48.1	Didelphidae: *Marmosops* sp.	122.2	19.14	12.7
QCAZR-12590	Manabi	F	J	557	495	8.42	41.5	Echimydae: *Proechimys* sp.	113.04	15.59	10.8
								Cricetidae	n.a.	n.a.	3.2
QCAZR-12613	Chimborazo	F	J	562	490	7.33	49.46	Cricetidae: *Melanomys* sp.	127.65	24.45	20.54
								Cricetidae	n.a.	n.a.	1.18
QCAZR-12614	Chimborazo	F	S	948	831	12.12	262.05	Cricetidae: *Microryzomys* sp.	130	20.23	21.5
								Cricetidae: *Thomasomys* sp.	n.a.	n.a.	5.02
QCAZR-12615	Chimborazo	F	S	871	760	12.74	213.63	Cricetidae: *Microryzomys* sp.	109.3	23.43	17.73
								Cricetidae	n.a.	n.a.	7.1
QCAZR-12785	Azuay	F	A	1,724	1,516	24.05	1,578.2	Cricetidae: *Sigmodon peruanus*	210	n.a.	78.3
								Cricetidae	n.a.	n.a.	9.5
QCAZR-13135	Guayas	F	A	1,425	1,275	21.6	762.2	Cricetidae: *Oligoryzomys* sp.	n.a.	n.a.	6.8
								Mammalia	n.a.	n.a.	n.a.
QCAZR-13136	Guayas	F	J	382	332	5.8	17.5	Cricetidae	n.a.	n.a.	0.7

**Note:**

Museum #, Museum number. Sex: F, female; M, male. Size-class: J, juvenile; S, subadult; A, adult. TL, Total length; SVL, snout-vent length; ND, Neck diameter (all given in mm); Mass (g). Prey items: L, Prey length (mm); D: Prey diameter (mm); M, Prey mass (g).

We found a total of 69 identifiable prey items and four unidentifiable ones (Table 1). In terms of number of prey items encountered per snake, three specimens (6%) had three items each, 20 snakes (43%) had two items, while 24 snakes had one prey item each, for an average of 1.55 prey items per snake; there was no difference between size-classes (Kruskal–Wallis: *p* = 0.332). Each prey item was classified by the state of preservation in which it was found: complete prey = 22; incomplete prey = nine; residuals = 34. No adult pitviper contained complete prey items. According to our taxonomic grouping, prey included six centipedes (Scolopendromorpha), 11 frogs (Anura), three lizards (Squamata), and 49 mammals (Rodentia and Didelphimorphia) (Table 1). The prey was generally ingested headfirst (96.8%). Centipedes were swallowed folded in two, with a single bite in the first third of the body (QCAZ 5760, 12463).

The analysis of occurrence of prey categories indicated that models based on both snake size and sex were the best fit to determine the presence of frogs, lizards, and mammals in the diet of *B. asper*, whereas the presence of centipedes was best predicted by a model based on SVL alone (Table 2). The sex of the snake was not a significant predictor of the prey taxa it consumed (Table 3), and only the SVL had a significant effect on the probability that a snake consumed amphibians and mammals. Specifically, we observed an ontogenetic shift, showing that the probability that a snake consumes amphibians is reduced and the probability that it eats mammals increases as the snake increases in size (Table 3).

**Table 2:** Model selection statistics.

Response variable	Model	d.f.	AIC_c_	ΔAIC_c_	Weight
Scolopendromorpha	**~SVL**	**2**	**28.81**	**0.00**	**0.38**
	~Null	1	28.90	0.09	0.36
	~Sex	2	30.87	2.06	0.14
	~Sex + SVL	3	31.10	2.29	0.12
Anura	**~Sex + SVL**	**3**	**25.38**	**0.00**	**0.57**
	~SVL	2	25.92	0.55	0.43
	~Sex	2	39.15	13.77	0.00
	~Null	1	40.65	15.28	0.00
Squamata	**~Sex + SVL**	**3**	**16.23**	**0.00**	**0.42**
	~SVL	2	16.94	0.71	0.30
	~Null	1	18.37	2.14	0.15
	~Sex	2	18.55	2.32	0.13
Mammalia	**~Sex + SVL**	**3**	**28.32**	**0.00**	**0.52**
	~SVL	2	28.50	0.19	0.48
	~Sex	2	45.20	16.89	0.00
	~Null	1	46.68	18.36	0.00

**Note:**

GLMs with a binomial distribution evaluating the frequency of occurrence (presence/absence) of the main taxonomic categories in the diet of *Bothrops asper*, as a function of the sex and SVL of the snake. Chosen models appear in bold. AICc, corrected Akaike Information Criterion.

**Table 3:** Summary of the best-fitting generalized binomial models (evaluated using the corrected Akaike information criterion) that assess the effect of snake sex and SVL on the frequency of occurrence of the main taxonomic categories in the diet of *Bothrops asper*.

	Predictor	Estimate	SE	Odds ratio	*z*	*p*
Scolopendromorpha						
	Intercept	−2.3026	0.5244	–	−4.3909	0.689
	SVL	−0.0028	0.0022	−0.28	−1.281	0.2
Anura						
	Intercept	4.0245	2.2452	--	1.792	0.0731
	Sex—male	1.9816	1.2811	625.43	1.547	0.1219
	**SVL**	−0.0139	0.0058	−1.38	−2.409	**0.0160**
Squamata						
	Intercept	1.9421	2.6812	–	0.7244	0.4688
	Sex—male	−18.5301	3911.2674	−99.99	−0.0047	0.9962
	SVL	−0.0083	0.0064	−0.83	−1.3091	0.1905
Mammalia						
	Intercept	−4.3263	2.0329	–	−2.1282	0.0333
	Sex—male	−1.7229	1.1755	−82.15	−1.4656	0.1428
	**SVL**	0.0128	0.0048	1.28	2.6593	**0.0078**

**Note:**

In bold, the factors with a significant effect. SE, standard error.

**Figure 1:**
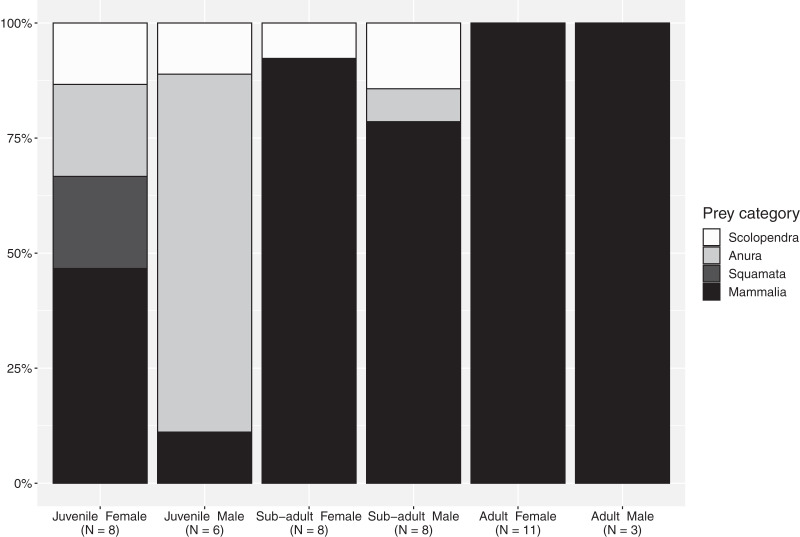
Percentages of the main prey categories from each sex and size class in the diet of *Bothrops asper* from Ecuador (non-identifiable preys removed).

The importance index showed that mammals represented the most important prey type (*I*_Mammalia_ = 76.9), in regard to all evaluated factors—abundance, frequency of occurrence and mass—while amphibians, centipedes and reptiles represent occasional meals with smaller contribution in particular in terms of mass (Table 4). The proportion of mammals increases from juveniles to subadults and reaches maximum values in adults, although this food type represented a considerable portion of the diet for all size-classes (Fig. 1). Spiny rats (Echimydae) were common in the diet of *B. asper* (*n* = 4; Table 1); the GIT of one adult female (QCAZ 5858) contained spines with an average length of 10 cm from such a rat (*Proechimys semiespinosus*). The niche breadth values were slightly lower in mature individuals (Simpson’s *B* juveniles: 0.885; sub-adults: 0.894; adults: 0.778). Juvenile males consumed significantly more ectotherms (mainly amphibians) than juvenile females (χ^2^ = 8.5, d.f. = 3, *p* = 0.04; Fig. 1), but no differences were found between the other size classes. Diversity indices were identical for the two sexes (Simpson’s *B* = 0.89).

**Table 4:** Importance index (*I_ x_*) from the composition of prey found in *Bothrops asper* from Ecuador.

	*n*_*x*_	*n*_*x*_ *%*	*f*_*x*_	*f*_*x*_ *%*	*m*_*x*_	*m*_*x*_ *%*	*I*_*x*_
Scolopendromorpha	6	8.7	4	8.3	10.2	3.7	6.9
Anura	11	15.9	7	14.6	15.9	5.7	12.1
Squamata	3	4.3	2	4.2	9.9	3.6	4.03
Mammalia	49	71.01	35	72.9	240.2	86.9	76.9

**Note:**

*n_x_*, abundance of prey item by taxonomic classification; *f_x_*, frequency of the type of prey *x*; *m_x_*, mass of each complete prey item by taxonomic classification. %, percentage for each category. Abundance and frequency indexes calculated based on all identifiable prey items (*n* = 69), mass based on complete preys only (*n* = 22). Adapted from Biavati, Wiederhecker & Colli (2004).

**Figure 2:**
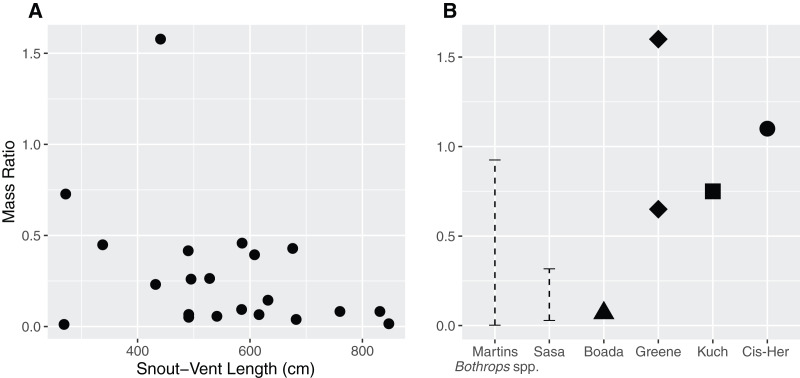
Relationship between the size of the snake (Snout–Vent Length) and the mass ratio (MR = prey mass/snake mass) (A) for *Bothrops asper* in Ecuador. The analysis was carried out only for cases where total prey mass could be precisely measured (*n* = 22). (B) For comparison, other reported mass ratios from literature: Martins–MR range between 0.002–0.925 based on data for several *Bothrops* species from Brazil (Martins, Marques & Sazima, 2002); Sasa—MR range between 0.029–0.317, obtained from *B. asper* in Costa Rica (Sasa, Wasko & Lamar, 2009); Boada—MR = 0.07, report of a single complete prey of *B. asper* from Manabí, Ecuador (Boada et al., 2005); Kuch—two MR values (>0.25 partially digested and 0.75) from *B. asper* in Ecuador (Kuch et al., 2004); Greene—MR = 0.65 for *B. asper* and MR = 1.6 for *B. atrox* both from Colombia (Greene, 1992); Cis-Her—MR = 1.1, data for *B. asper* from southern Ecuador (Cisneros-Heredia, 2006).

The mass ratio in our sample ranged between 0.011–0.727, with an extreme value of 1.577 (Fig. 2A) in the case of a juvenile female (QCAZ 5879) containing a mist rodent (*Nephelomys moerex*). Mass ratio was not influenced by either sex (*p* = 0.07) or SVL (*p* = 0.15) of the snake (*F*_2,19_ = 2.72, adjusted *r*^2^ = 0.14). Similarly, the model investigating the ratio between the prey’s largest width and the snake neck diameter indicated that neither sex (*p* = 0.2) nor SVL (*p* = 0.83) influenced the DR (*F*_2,19_ = 0.92, adjusted *r*^2^ = 0).

### Bibliographic research

**Figure 3:**
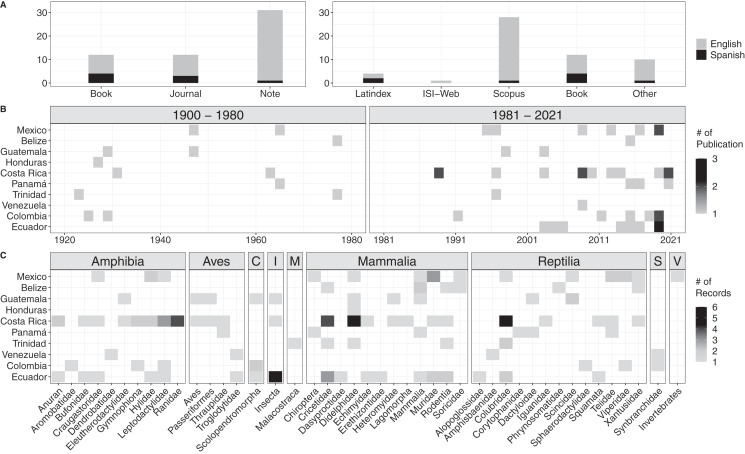
Results of the bibliographic search of the diet of Tercioppelos (*Bothrops asper*). (A) Quantification of publication method and accessibility. Other: papers not indexed in Web of Science, Latinidex or Scopus. (B) Number of publications by year and country. (C) Number of records by country for each taxonomic group. C, Chilopoda; I, Insecta; M, Malacostraca; S, Synbranchidae; V, Unspecified invertebrate.

We encountered a total of 54 bibliographic accounts for prey data of Tercipelos (Fig. 3, Table S1). We were not able to access the full text of seven of them (older, out of print books, not available through any digital repository), but we include them since their contents regarding the Terciopelo diet was cited in previous bibliographic reviews or publications. Most diet records were accessible through the most prominent search databases (Fig. 3A). Diet data were available for 10 of the 12 countries in which this species is present, except for Nicaragua and Peru (Fig. 3B). The prey types were classified into nine classes, with 38 subclasses (mainly Families) and 134 individual records (Fig. 3C, Table S2). Terciopelo feeding habits in literature were mainly published as short notes (*n* = 56.4%), and most were written in English (*n* = 85.5%). Of the encountered records, 18.2% were not available through online search methods, most of these in Spanish. Most of the records come from the last 40 years (76.4%); the establishment of new research stations and scientific collections in the Neotropics, such as La Selva Biological Station, Instituto Clodomiro Picado and the Museum of Zoology QCAZ have contributed to the rise in available information (Costa Rica: 44, Ecuador: 27, representing 53% of the total available data). Mammals represented 47 records, followed by reptiles (34) and amphibians (30). However, 18% of the prey records came from less typical prey items, such as invertebrates, centipedes, crayfish and fish.

## Discussion

### Prey diversity

Following the definition of Martins, Marques & Sazima (2002), a diet specialist should have at least 75% of its diet composed of one particular prey type. Our results concur with previous studies that show *B. asper* to be an overall generalist (Sasa, Wasko & Lamar, 2009) and a highly adaptable predator (Fig. 1), capable of consuming a diverse range of prey types throughout its life (Table 1). However, when looking specifically at various size-classes, our data indicate that subadults and adults should be considered mammal specialists. Nevertheless, although few food items listed in previous literature provide information related to predator sex and size-class (*n* = 20/134 of individual records) or only size-class (*n* = 83/134 of individual records), when added to our findings, they confirm an unequal prey use in these vipers (Figs. S1, S2). Terciopelo diet included a variety of challenging prey items: aggressive and venomous centipedes (where the snake could become the prey, especially in juveniles), heavily spined mammals, toxic frogs, and venom resistant marsupials (Tables 1 and S2, Figs. S3–S6).

Most of the reports of *B. asper* diet are based on small sample sizes. The only two other studies based on a larger number of specimens—Boada et al. (2005) with 21 specimens inspected, 14 with GIT content, and Sasa, Wasko & Lamar (2009) with 56 specimens inspected, 32 with GIT content—indicate that it is common for the inspected snakes to not have identifiable prey items. This was certainly the case in our study, where we found that 24.4% of specimens contained some form of prey item (193 inspected, 47 with GIT content), with an average of 1.5 prey item per GIT. The aforementioned large-scale studies also gave us a measure of how frequently different taxonomic groups are taken, reflected by the proportion of snakes that presented them in their GIT: 64.3% insects, 42.9% mammals, 21.4% anurans, 7.1% birds, 7.1% lizards and 7.1% scolopendras in the Terciopelos from Ecuador (Boada et al., 2005) and 69% mammals, 10.3% birds, 10.3% lizards, 6.9% anurans and 3.4% orthopterans for the snakes from Costa Rica (Sasa, Wasko & Lamar, 2009). In our case, mammals (71%) dominated the diet of *B. asper*, and amphibians (15.9%) and scolopendras (8.7%) represented an important component in the diet, especially in early stages. The number of reptiles (4.3%) and birds (0%) is lower in our sample compared to these studies (Table 1). Given the large distribution of Terciopelos and the variety of habitats they occupy, the differences in diet might be due to habitat composition and prey availability. The abundance of some amphibians and reptiles in their habitat also changes with seasons, and this in turn affects their proportion in the predator diet (Shine, 1994; Greene, 1997; Martins, Marques & Sazima, 2002). Consequently, these differences should be reflected in the diet of the snakes. For example, large species of frogs (*e.g*., *Leptodactylus*) or lizards (*e.g*., *Basiliscus* and *Ctenosaura*) have been reported as prey of Terciopelos in adults (Sasa, Wasko & Lamar, 2009; Wasko & Sasa, 2012; Fortier, 2021), which may relate to their abundance in different areas that may vary temporally. On the other hand, birds seem to be a more difficult prey to catch and a less common prey for most *Bothrops* species. This is due not only to the differences in microhabitat use, and therefore fewer hunting opportunities, but also to the fact that hunting birds may require venom specialization (Hayes, 1992; Martins, Marques & Sazima, 2002). The prevalence of mammals as prey items might be the result of hunting microhabitat preference by sex, and consuming mammals has been shown to enhance the fitness of offspring in *Bothrops* and other vipers (Martins, Marques & Sazima, 2002). Adding to this multidimensional question is the fact that the preferred habitat of Terciopelos seems to be early succession shrubby forest, and consequently they are expected to eat mostly prey species associated with intervened forest (Sasa, Wasko & Lamar, 2009; Hernández-Ordóñez, Urbina-Cardona & Martínez-Ramos, 2015; Ramírez-Arce, Zúñiga-Ortiz & Wasko, 2021). Prey composition may be notably affected by life-stage and sex; however, due to the difficulty of collecting enough samples from different biomes, such relations remain unclear (Urbina-Cardona, Olivares-Pérez & Reynoso, 2006). Ultimately, the lack of diversity displayed in the diet of adults in our data set could be the result of a low sample size, as shown by a better sampled population of adults in Costa Rica (Sasa, Wasko & Lamar, 2009).

We found that SVL was the main predictor for the probability that a Terciopelo consumes amphibians and mammals; as the snakes increase in size, the probability that they consume amphibians decreases, in favor of mammals. This ontogenetic transition from ectotherm to endotherm prey has been previously mentioned (Picado, 1931; Solórzano, 2004; Sasa, Wasko & Lamar, 2009), and this is accompanied by a shift in venom composition (Sasa, Wasko & Lamar, 2009; Mora-Obando et al., 2020). For reptiles or centipedes, SVL had no significant effect, which we attribute to the low sample size, as these prey types are taken in an opportunistic manner.

Terciopelos can thrive in anthropogenic ecosystems (Otero-Patiño, 2009; Sasa, Wasko & Lamar, 2009; Chippaux, 2017), hunting near human settlements and feeding on their pests (Boada et al., 2005), which is supported by our findings of 23% of specimens (45/193) having damaged heads—likely due to negative human encounters. Yet, Wasko & Sasa (2010, 2012) and Ramírez-Arce, Zúñiga-Ortiz & Wasko (2021) show that *B. asper* tends to actively avoid human interaction and settlements, suggesting their relationship with anthropogenic areas as hunting grounds may require further investigation. While some individuals were found in the vicinity of La Selva Biological Station (Costa Rica), the authors note that the circumstances that motivate these incursions are unknown. Our findings of diet support this observation, since the species of centipedes and amphibians (*e.g*., *Leptodactylus* sp., *Pristimantis* sp.) that we registered in the GIT contents are associated with modified areas (Ron, Merino-Viteri & Ortiz, 2022). We suspect that such prey is mostly taken by opportunistic juveniles without an established hunting ground. On the other hand, the mammal species which formed part of the diet (*e.g*., *Melanomys* sp., *Proechimys* sp.) are mostly typical for primary or secondary forest (Sasa, Wasko & Lamar, 2009; Brito, Camacho & Vallejo, 2022). This suggests that adult Terciopelos found near human settlements might have been displaced from their original hunting ground, rather than actively preferring anthropogenic habitats. Although there is no doubt that some Terciopelos find anthropogenic habitats suitable for hunting (Ramírez-Arce, Zúñiga-Ortiz & Wasko, 2021), our results, as well as the literature records, suggest that generally these vipers tend to avoid them.

### Predator-prey size relationship

The size of the snake was not a good predictor for the size of the prey they ingested. The MR values found in our study (Fig. 2A; ranging between 0.011–0.727, with an outlier of 1.577) are consistent with previous observations (Fig. 2B) for this species—Costa Rica (0.029–0.317; Sasa, Wasko & Lamar, 2009), Colombia (0.65; Greene, 1992), Ecuador (0.75–1.1; Boada et al., 2005, 0.07; Kuch et al., 2004; Cisneros-Heredia, 2006). Other *Bothrops* species from Brazil have MR ranging between 0.002–0.925 (Martins, Marques & Sazima, 2002), although there are also reports of more copious meals (Greene, 1992; the present study). An ample review from Glaudas et al. (2019) including 129 snake taxa reports MR ranges of 0.00–1.67 (this being the largest MR reported for snakes). Overall, these works indicate that ambush predators do not specialize in larger prey items, but rather are opportunistic and capable of consuming large prey when given the chance. This is consistent with our findings and prey items reported for *B. asper*.

An increase in MR is often theorized due to the ontogenetic shift from ectothermic prey to endotherms, since taken frogs, lizards, and scolopendras tend to have lower mass than mammals (Table 4, Martins, Marques & Sazima, 2002; Sasa, Wasko & Lamar, 2009). Although this pattern was observed by Sasa, Wasko & Lamar (2009), they attribute this trend to a low sample size. Martins, Marques & Sazima (2002) and Nogueira, Sawaya & Martins (2003) encountered either the inverse pattern or no trend at all in several well sampled species of *Bothrops* from Brazil, while Glaudas et al. (2019) found no such tendency in ambush predators and a decrease of MR in active foragers. Even without records of complete food items in adults, our data do not show this trend. However, the MR is likely to be highly dependent on the characteristics of the available prey; as such, the amphibians and reptiles reported in the diet of *B. asper* from Central America are of considerable mass (Sasa, Wasko & Lamar, 2009; Fortier, 2021). Additionally, Terciopelo individuals can consume several food items in short periods, so the total mass they ingest can be quite large, even if the individual items are small, which is a common tendency in ambush foragers (Glaudas et al., 2019).

### Prey item notes

A female juvenile (QCAZ 12463) had in its stomach a Roof Anole (*Anolis bitectus*) and a centipede, side by side, both showing one bite in the middle of the body. The state of digestion of both specimens was minor, which meant that both kills must have happened recently (Fig. S4). While the separate MR of these items (0.05 and 0.066 respectively) are not remarkable, together they make a considerable meal (MR 0.117). Furthermore, this record supports the idea of the opportunistic nature of Terciopelos, which will eat, if given the chance, several small items in short periods of time. Additionally, Terciopelos seem to catch long but narrow prey items by a single bite in the middle of the prey’s body (Jones, Straka & Kayano, 2014). It is unclear if this strategy is learned, although there seems to be trial and error involved (see Ryan et al., 2010; Díaz-Ricaurte, 2018; Szczygieł & Page, 2020).

The outlier MR record (1.577) found in our sample came from a juvenile female (QCAZ 5879), which ingested an adult mist rodent (*Nephelomys moerex*) measuring 166 mm in length (37% of the snake SVL). This is one of the highest reported values that we encountered in literature for the species. A similar value (MR = 1.6) reported in its sister species, *Bothrops atrox*, by Greene (1992) consisted of a snake that had eaten a whiptail lizard (*Cnemidophorus gramivagus*) in Colombia.

An adult female (QCAZ 5858) had in its stomach the remains of a spiny rat (*Proechimys semiespinosus*). However, the state of digestion in which it was found was very advanced, with no soft tissue left, and only the complete skeleton and spines remaining (Fig. S5). No remains were found in the intestines or rectum. This record is remarkable for two reasons. First, the spines were large, and they had perforated part of the stomach and epithelium of the snake; however, this could have happened after the death of the snake or during the preservation of the specimen. Second, this female was the only one of the snakes we examined with gut contents where we observed developing follicles. She had 19 embryos (the biggest one being 32 mm in diameter), all in an advanced stage of development (stage 37 according to Zehr, 1962). The lack of soft tissue indicates that the prey had been consumed during early stages of embryonic development and the snake stopped all GIT movement (Secor, 2005). Such post-prandial reduction of the activity in the digestive system has been previously reported in ambush-foraging predators, and it is attributed to energy saving (Secor & Diamond, 1998; Glaudas et al., 2019). This is a poorly discussed phenomenon during reproduction stages in snakes, and we recommend future investigation examine it further.

### Sexual dimorphism and diet

Our results show that the sex of the snake influences the type of prey it consumes at least in the juvenile size-class, a pattern similar to the one reported by Sasa, Wasko & Lamar (2009), although they only based their conclusions on adults. In our sample, while sex did not affect the probability that the snake consumed certain taxa, and the detailed diversity index found similar values for males and females, differences appear when considering the proportions for various prey categories, revealing a tendency for juvenile females’ diet to consist of more mammals than the diet of juvenile males. A similar pattern is shown by the literature records for which sex and size class are available (Table 1, Figs. S1, S2).

Sexual dimorphism in *B. asper* has been well-documented, females being larger than males (Sasa, 2002; Saldarriaga-Córdoba et al., 2009; Sasa, Wasko & Lamar, 2009; Henao-Duque & Ceballos, 2013; Gómez et al., 2021). This might on its own explain the mammal predominance in the females’ diet. However, other factors might cause the observed differences in the diet of the two sexes. For example, in juveniles, although tail coloration is sexually dimorphic (males having bright yellow tails), both sexes exhibit caudal-luring behavior (Neill, 1960; Tryon, 1985; Solórzano & Cerdas, 1989), however the effectiveness of this behavior relating the color variation has not been tested. Venom composition, geographic and ontogenetic venom variation are well documented in the species (Lomonte & Carmona, 1992; Saldarriaga et al., 2003; Alape-Girón et al., 2008; Alape-Girón et al., 2009; Mora-Obando et al., 2020; Gómez et al., 2021), with no differences in venom between the sexes in adults (Gómez et al., 2021). Yet, it is not known if there are venom differences between sexes in juveniles. Spatial ecology and habitat preferences tested in populations from Costa Rica have found no differences overall between sexes; however, there are significant differences between males and females in distance to the nearest body of water and microhabitat usage between daytime and nighttime, although, again, only in adults (Wasko & Sasa, 2010). Since there are no documented male-male combat events in Terciopelos (Sasa, Wasko & Lamar, 2009), and there seem to be differences in sex ratios between populations (Savage, 2002; Wasko & Sasa, 2010; Ramírez-Arce, Zúñiga-Ortiz & Wasko, 2021; 105 females *vs*. 88 males in our study), the niche partitioning between sexes could be a possible explanation for differences in diet. This would allow enhanced fecundity in females, while males would take advantage of alternative prey species with less competition (Shine, 1994). Hence, differences in head allometry and/or behavior could be crucial in understanding the differences in diets between sexes (Sasa, Wasko & Lamar, 2009). Additionally, sexual dimorphism in *B. asper* should be studied at a population level, due to the high diversity within this species complex (Shine & Goiran, 2021). It is likely that sexual dimorphism is dependent on macrohabitat conditions and heavily influenced by speciation (Hendry, Guiher & Pyron, 2014), which occurs in many Terciopelo populations (Saldarriaga-Córdoba et al., 2009; Salazar-Valenzuela, 2016; Saldarriaga-Córdoba et al., 2017; Salazar-Valenzuela et al., 2019; Mora-Obando et al., 2020).

### Data regarding Terciopelo prey records in literature

The confusion caused by the use of common names is one of the reasons why the binomial nomenclature proposed by Linné is considered crucial in natural sciences (Bennett & Balick, 2014). In our review, we aimed to clarify some inaccuracies present in previous works (Sasa, Wasko & Lamar, 2009; Farr & Lazcano, 2017). Such is the case of a record originally listed as “zorrillos” by Picado (1931) and invalidly cited afterwards by Farr & Lazcano (2017) as “skunks” (“zorrillos” is commonly used as a synonym of marsupials; M. Sasa, 2021, personal communication).

One interesting aspect is that *B. asper* diet contains a rather insignificant number of species which are commonly considered as human-associated (*e.g*., *Rattus rattus* and *Mus musculus*). This suggests that *B. asper* avoids human vicinities, as supported also by telemetry data (Wasko & Sasa, 2010, 2012; Ramírez-Arce, Zúñiga-Ortiz & Wasko, 2021).

Since the first diet records of the Terciopelos, possums have been listed as prey items (Mole, 1924; Picado, 1931). Sadly, many of the names used are generic and do not provide a clear taxonomic identification from this diverse group (Voss & Jansa, 2009). Few and far between, they are extremely important from an evolutionary point of view, since some species of possums are resistant to venoms from pitvipers (Voss & Jansa, 2012). Voss (2013) revisits some of these records and develops a poorly explored topic in viper ecology and evolution.

It is still unclear if insects are an intentional part of the diet of Terciopelos. Boada et al. (2005) and Sasa, Wasko & Lamar (2009) list insects as prey items, because juveniles might use large grasshoppers and beetles as prey. However, it is generally assumed that insect remains are secondary or unintentional prey, and this appears to be the case in our study, since all the remains came from small insects found in the hindgut.

The importance of methodical and systematic literature searches for previous publications cannot be disregarded. This is especially the case for species with the taxonomic and phylogenetic complexity of *Bothrops*, which has captured the attention of academics for hundreds of years. The hunt for these records must take into account old names in the different locations of distribution and taxonomic history. Researchers working in different parts of the distribution of a taxon must aim to cite local (peer-reviewed) papers that would be difficult for foreign investigators to find. The Internet and many of the research databases are important tools to find such records (for detailed recommendations, see van den Burg, 2020; Maritz et al., 2021). However, a consequence of the search algorithms, language barriers and skewed impact metrics is that sometimes important regional and localized reports are less likely to be found (Gasparyan et al., 2017). Research communities are a valuable way to put forward such reports and ensure that primary data from, and throughout, a species range are incorporated into analyses and given weight according to their quality, not age, language, or publication outlet (Maritz et al., 2021).

Finally, we recommend that the researchers report the highest level of details available. In our case, as discussed before, the identification of the items was done to the lowest taxonomic level possible. Additionally, predator sex, size of prey and predator (diameter, length, and mass), state of digestion, location in the GIT, and pictures (considering the current easy access to technology) should be recorded whenever possible (for a detailed guide see Maritz et al., 2021). This could provide additional details which can be used in reviews and metanalyses in the future (*e.g*., sexual dimorphism influence on the diet). Usually, museums have difficulties maintaining large collections of the same species, especially with large sized specimens, but the preservation of these kind of samples could yield particularly important results for future unforeseen methodologies (Glaudas et al., 2019).

### Bias in reporting

Short dietary scientific notes are crucial to provide additional information about the breadth of the diet of a species (Fig. S7). However, the tendency to report rare and sensational prey items can lead to biases when evaluating the proportions of different prey types between sexes and/or age/size class (Maritz et al., 2021). In addition, the use of GIT samples from museum specimens can have its own biases (Glaudas, Kearney & Alexander, 2017), such as lower detection of certain types of prey. For example, amphibians, which are of smaller size and rapidly digested, are likely to be overlooked, so their representation in the diet analysis can be underestimated. This could also affect our sample, since there was a 5% (*i.e*., four items) of unidentifiable stomach content, consisting of organic material in the small intestine that we were unable to identify. The only way to reduce the bias in reporting would be to systematically collect data from different population, demographics, and seasons, which would allow a better understanding of the niche that this species occupies and the role that it plays in the ecosystem, depending on its sex and life-stage.

## Conclusions

The diet of the Terciopelo pitviper reflects the diverse habitats it inhabits, supporting the idea of an opportunistic predator. The size of the snake had a strong influence on the type of prey it eats in early stages, but less so on the size of its prey. When looking at the composition of the diet, the lack of prey items associated with humans suggests that *B. asper* tends to avoid anthropogenic areas and the individuals found near settlements are likely ones who have been displaced from their original hunting grounds. Data made available through short scientific notes, packed with as much information as possible, is crucial to provide a better understanding of the dietary diversity of this widespread species and its role in the ecosystem. However, systematic collection of information from different populations, demographics, and seasons is necessary to determine the ontogenetic, sexual and geographic variability in the feeding habits of this species complex. Additional studies are necessary to understand how sexual morphometrics influences the role and selective pressures in this species.

## Supplemental Information

10.7717/peerj.14817/supp-1 Supplemental Information 1 Percentages of the main prey categories available in literature (Table 5) throughout the distribution of *Bothrops asper* from where sex and size class are available.

10.7717/peerj.14817/supp-2 Supplemental Information 2 Percentages of the main prey categories available in literature (Table 5) throughout the distribution of *Bothrops asper* from where only size class is available.

10.7717/peerj.14817/supp-3 Supplemental Information 3 A male sub-adult (QCAZ 11617) showcasing a considerable sized rat (MR 0.43).

10.7717/peerj.14817/supp-4 Supplemental Information 4 A female juvenile (QCAZ 12463) with two prey items side by side in its stomach, a Roof Anole (*Anolis bitectus*) and a centipede.

10.7717/peerj.14817/supp-5 Supplemental Information 5 GIT of adult female (QCAZ 5858) containing spines with an average length of 10 cm from a spined rat (Proechimys semiespinosus) alongside its well-developed embryos.

10.7717/peerj.14817/supp-6 Supplemental Information 6 A male sub-adult (QCAZ 5760) with a centipede. Showcasing the swallow method by folding long prey.

10.7717/peerj.14817/supp-7 Supplemental Information 7 Percentages of the main prey categories available in literature (Table 5) throughout the distribution of *Bothrops asper* categorized by country.

10.7717/peerj.14817/supp-8 Supplemental Information 8 Compilation of literature publications with records of diet of *Bothrops asper* throughout its distribution.

10.7717/peerj.14817/supp-9 Supplemental Information 9 Prey taxa reported from the diet of *Bothrops asper* throughout its distribution.

10.7717/peerj.14817/supp-10 Supplemental Information 10 Raw data.

## Additional Information and Declarations

### Data Availability

The following information was supplied regarding data availability: The raw data and information regarding museum specimen number, characteristics, and full details of their prey are available in the Supplemental Files.

## References

[ref-1] Alape-Girón A, Flores-Díaz M, Sanz L, Madrigal M, Escolano J, Sasa M, Calvete JJ (2009). Studies on the venom proteome of *Bothrops asper*: perspectives and applications. Toxicon.

[ref-2] Alape-Girón A, Sanz L, Escolano J, Flores-Diaz M, Madrigal M, Sasa M, Calvete JJ (2008). Snake venomics of the lancehead pitviper *Bothrops asper*: geographic, individual, and ontogenetic variations. Journal of Proteome Research.

[ref-4] Barbour T, Loveridge A (1929). On *Bothrops atrox* (Linné). Bulletin of the Antivenin Institute of America.

[ref-5] Bennett BC, Balick MJ (2014). Does the name really matter? The importance of botanical nomenclature and plant taxonomy in biomedical research. Journal of Ethnopharmacology.

[ref-6] Biavati GM, Wiederhecker HC, Colli GR (2004). Diet of *Epipedobates flavopictus* (Anura: Dendrobatidae) in a neotropical savanna. Journal of Herpetology.

[ref-7] Boada C, Salazar-Valenzuela D, Lascano A, Kuch U (2005). The diet of *Bothrops asper* (Garman, 1884) in the Pacific lowlands of Ecuador. Herpetozoa.

[ref-8] Brito J, Camacho MA, Vallejo AF (2022). Mamíferos del Ecuador. Versión 2022.0. Museo de Zoología, Pontificia Universidad Católica del Ecuador. https://bioweb.bio/faunaweb/mammaliaweb/.

[ref-9] Burnham KP, Anderson DR (2004). Multimodel inference: understanding AIC and BIC in model selection. Sociological Methods & Research.

[ref-14] Campbell JA, Lamar WW (2004). The venomous reptiles of the western hemisphere.

[ref-16] Chippaux J-P (2017). Incidence and mortality due to snakebite in the Americas. PLOS Neglected Tropical Diseases.

[ref-17] Cisneros-Heredia DF (2006). Distribution and ecology of the western Ecuador frog *Leptodactylus labrosus* (Amphibia: Anura: Leptodactylidae). Zoological Research.

[ref-18] Cisneros-Heredia DF, Touzet JM (2004). Distribution and conservation status of *Bothrops asper* (Garman, 1884) in Ecuador. Herpetozoa.

[ref-19] Díaz-Ricaurte JC (2018). First record of attempted piscivory by *Bothrops asper* (Garman, 1883)(Squamata, Viperidae) on a swamp eel, genus *Synbranchus*. Herpetology Notes.

[ref-21] Farr WL, Lazcano D (2017). Distribution of *Bothrops asper* in Tamaulipas, Mexico and a review of prey items. The Southwestern Naturalist.

[ref-22] Feldman A, Meiri S (2013). Length-mass allometry in snakes. Biological Journal of the Linnean Society.

[ref-23] Fortier R (2021). *Bothrops asper* (Fer-de-Lance). Diet. Herpetological Review.

[ref-26] Gasparyan AY, Nurmashev B, Yessirkepov M, Endovitskiy DA, Voronov AA, Kitas GD (2017). Researcher and author profiles: opportunities, advantages, and limitations. Journal of Korean Medical Science.

[ref-27] Gibert JP, DeLong JP (2017). Phenotypic variation explains food web structural patterns. Proceedings of the National Academy of Sciences of the United States of America.

[ref-28] Glaudas X, Glennon KL, Martins M, Luiselli L, Fearn S, Trembath DF, Jelić D, Alexander GJ (2019). Foraging mode, relative prey size and diet breadth: a phylogenetically explicit analysis of snake feeding ecology. Journal of Animal Ecology.

[ref-29] Glaudas X, Kearney TC, Alexander GJ (2017). Museum specimens bias measures of snake diet: a case study using the ambush-foraging puff adder (*Bitis arietans*). Herpetologica.

[ref-30] Grainger R, Peddemors VM, Raubenheimer D, Machovsky-Capuska GE (2020). Diet composition and nutritional niche breadth variability in juvenile white sharks (*Carcharodon carcharias*). Frontiers in Marine Science.

[ref-31] Greene HW (1983). Dietary correlates of the origin and radiation of snakes. American Zoologist.

[ref-32] Greene HW, Greene HW, Campbell JA, Brodie ED (1992). The ecological and behavioral context for pitviper evolution. Biology of the Pitvipers.

[ref-33] Greene HW (1997). Snakes: the evolution of mystery in nature.

[ref-35] Gómez A, Segura Á, Solano G, Chacón D, Corrales G (2021). Influence of sexual dimorphism on venom composition in *Bothrops asper* and *Crotalus simus* (Serpentes: Viperidae) and its potential implications on the snake antivenom production. Toxicon.

[ref-36] Hayes WK (1992). Prey-handling and envenomation strategies of prairie rattlesnakes (*Crotalus v. viridis*) feeding on mice and sparrows. Journal of Herpetology.

[ref-37] Henao-Duque AM, Ceballos CP (2013). Sex-related head size and shape dimorphism in Mapaná snakes (*Bothrops asper*) kept in captivity. Revista Colombiana de Ciencias Pecuarias.

[ref-39] Hendry CR, Guiher TJ, Pyron RA (2014). Ecological divergence and sexual selection drive sexual size dimorphism in New World pitvipers (Serpentes: Viperidae). Journal of Evolutionary Biology.

[ref-40] Hernández-Ordóñez O, Urbina-Cardona N, Martínez-Ramos M (2015). Recovery of amphibian and reptile assemblages during old-field succession of tropical rain forests. Biotropica.

[ref-43] Jones MA, Straka JR, Kayano K (2014). *Bothrops asper* (Fer-de-Lance). Diet. Herpetological Review.

[ref-44] King RB (1989). Sexual dimorphism in snake tail length: sexual selection, natural selection, or morphological constraint?. Biological Journal of the Linnean Society.

[ref-45] Kuch U, Boada C, García F, Torres J, Freire A (2004). *Bothrops asper* (Terciopelo or equis). Diet. Herpetological Review.

[ref-48] Lomonte B, Carmona E (1992). Individual expression patterns of myotoxin isoforms in the venom of the snake *Bothrops asper*. Comparative Biochemistry and Physiology Part B: Biochemistry and Molecular Biology.

[ref-50] March DDH (1928). Field notes on Barba amarilla (*Bothrops atrox*). Bulletin of the Antivenin Institute of America.

[ref-51] Maritz B, Hofmann E, Maritz R, Greene H, Grundler M, Durso A (2021). Challenges and opportunities in the study of snake diets. Herpetological Review.

[ref-52] Martins M, Gordo M (1993). *Bothrops atrox* (common lancehead). Diet. Herpetological Review.

[ref-53] Martins M, Marques OAV, Sazima I, Schuett GW, Höggren M, Douglas ME, Greene HW (2002). Ecological and phylogenetic correlates of feeding habits in Neotropical pitvipers of the genus *Bothrops*. Biology of the Vipers.

[ref-55] Mole RR (1924). The Trinidad snakes. Proceedings of the Zoological Society of London.

[ref-57] Mora-Obando D, Salazar-Valenzuela D, Pla D, Lomonte B, Guerrero-Vargas JA, Ayerbe S, Gibbs HL, Calvete JJ (2020). Venom variation in *Bothrops asper* lineages from North-Western South America. Journal of Proteomics.

[ref-59] Neill WT (1960). The caudal lure of various juvenile snakes. Quarterly Journal of the Florida Academy of Sciences.

[ref-60] Nicéforo-María H (1930). Los reptiles y batracios de Honda (Tolima) en el Museo de La Salle. Revista Sociedad Colombiana de Ciencias Naturales.

[ref-61] Nogueira C, Sawaya RJ, Martins M (2003). Ecology of the pitviper, *Bothrops moojeni*, in the Brazilian Cerrado. Journal of Herpetology.

[ref-63] Otero-Patiño R (2009). Epidemiological, clinical and therapeutic aspects of *Bothrops asper* bites. Toxicon.

[ref-64] Parker HW (1926). The reptiles and batrachians of Gorgona Island, Colombia. Annals and Magazine of Natural History.

[ref-65] Picado C (1931). Serpientes venenosas de Costa Rica.

[ref-67] Pough FH, Groves JD (1983). Specializations of the body form and food habits of snakes. American Zoologist.

[ref-68] R Core Team (2021). R: a language and environment for statistical computing.

[ref-69] Ramírez-Arce DG, Zúñiga-Ortiz A, Wasko DK (2021). Habitat use and age structure of the Fer-de-Lance (*Bothrops asper*, Viperidae) in Braulio Carrillo National Park, Costa Rica. Herpetological Journal.

[ref-70] Reuman DC, Cohen JE (2004). Trophic links’ length and slope in the Tuesday Lake food web with species’ body mass and numerical abundance: Trophic link lengths and slopes in a food web. Journal of Animal Ecology.

[ref-73] Ron SR, Merino-Viteri A, Ortiz DA (2022). Anfibios del Ecuador. Versión 2022.0. Museo de Zoología, Pontificia Universidad Católica del Ecuador. https://bioweb.bio/faunaweb/amphibiaweb.

[ref-74] Ryan M, Blea N, Latella I, Kull M (2010). *Leptodactylus savagei* (smoky jungle frog). Antipredator defense. Herpetological Review.

[ref-75] Salazar-Valenzuela D (2016). Diversification in the Neotropics: insights from demographic and phylogenetic patterns of lancehead pitvipers (*Bothrops* spp.).

[ref-76] Salazar-Valenzuela D, Kuch U, Torres-Carvajal O, Valencia JH, Gibbs HL (2019). Divergence of tropical pitvipers promoted by independent colonization events of dry montane Andean habitats. Journal of Biogeography.

[ref-77] Saldarriaga MM, Otero R, Núñez V, Toro MF, Díaz A, Gutiérrez JM (2003). Ontogenetic variability of *Bothrops atrox* and *Bothrops asper* snake venoms from Colombia. Toxicon.

[ref-78] Saldarriaga-Córdoba MM, Parkinson CL, Daza JM, Wüster W, Sasa M (2017). Phylogeography of the Central American lancehead *Bothrops asper* (Serpentes: Viperidae). PLOS ONE.

[ref-79] Saldarriaga-Córdoba MM, Sasa M, Pardo R, Méndez MA (2009). Phenotypic differences in a cryptic predator: factors influencing morphological variation in the terciopelo *Bothrops asper* (Garman, 1884; Serpentes: Viperidae). Toxicon.

[ref-80] Sanders KL, Rasmussen AR, Mumpuni, Elmberg J, de Silva A, Guinea ML, Lee MSY (2013). Recent rapid speciation and ecomorph divergence in Indo-Australian sea snakes. Molecular Ecology.

[ref-81] Sasa M (2002). Morphological variation in the lancehead pitviper *Bothrops asper* (Garman) (Serpentes: Viperidae) from Middle America. Revista de Biología Tropical.

[ref-82] Sasa M, Wasko DK, Lamar WW (2009). Natural history of the terciopelo *Bothrops asper* (Serpentes: Viperidae) in Costa Rica. Toxicon.

[ref-83] Savage JM (2002). The amphibians and reptiles of Costa Rica: a herpetofauna between two continents, between two seas.

[ref-84] Schalk CM, Cove MV (2018). Squamates as prey: predator diversity patterns and predator-prey size relationships. Food Webs.

[ref-85] Secor SM (2005). Evolutionary and cellular mechanisms regulating intestinal performance of amphibians and reptiles. Integrative and Comparative Biology.

[ref-86] Secor SM, Diamond J (1998). A vertebrate model of extreme physiological regulation. Nature.

[ref-89] Shine R (1991). Intersexual dietary divergence and the evolution of sexual dimorphism in snakes. The American Naturalist.

[ref-90] Shine R (1994). Allometric patterns in the ecology of Australian snakes. Copeia.

[ref-91] Shine R, Goiran C (2021). Sexual dimorphism in size and shape of the head in the sea snake *Emydocephalus annulatus* (Hydrophiinae, Elapidae). Scientific Reports.

[ref-92] Simpson EH (1949). Measurement of diversity. Nature.

[ref-93] Sivan J, Hadad S, Tesler I, Rosenstrauch A, Allan Degen A, Kam M (2020). Relative tail length correlates with body condition in male but not in female crowned leafnose snakes (*Lytorhynchus diadema*). Scientific Reports.

[ref-94] Smith HM (1947). Notes on Mexican amphibians and reptiles. Journal of the Washington Academy of Sciences.

[ref-95] Solórzano A (2004). Serpientes de Costa Rica: distribución, taxonomía e historia natural.

[ref-96] Solórzano A, Cerdas L (1989). Reproductive biology and distribution of the terciopelo, *Bothrops asper* Garman (Serpentes: Viperidae), in Costa Rica. Herpetologica.

[ref-99] Szczygieł H, Page R (2020). When the hunter becomes the hunted: foraging bat attacked by pit viper at frog chorus. Ecology.

[ref-100] Tryon BW (1985). *Bothrops asper* (terciopelo). Caudal luring. Herpetological Review.

[ref-102] Urbina-Cardona JN, Olivares-Pérez M, Reynoso VH (2006). Herpetofauna diversity and microenvironment correlates across a pasture–edge–interior ecotone in tropical rainforest fragments in the Los Tuxtlas Biosphere Reserve of Veracruz, Mexico. Biological Conservation.

[ref-104] van den Burg MP (2020). How to source and collate natural history information: a case study of reported prey items of *Erythrolamprus miliaris* (Linnaeus, 1758). Herpetology Notes.

[ref-107] Voris HK, Voris HH (1983). Feeding strategies in marine snakes: an analysis of evolutionary, morphological, behavioral and ecological relationships. American Zoologist.

[ref-108] Voss RS (2013). Opossums (Mammalia: Didelphidae) in the diets of Neotropical pitvipers (Serpentes: Crotalinae): evidence for alternative coevolutionary outcomes?. Toxicon.

[ref-109] Voss RS, Jansa SA (2009). Phylogenetic relationships and classification of Didelphid marsupials, an extant radiation of New World Metatherian mammals. Bulletin of the American Museum of Natural History.

[ref-110] Voss RS, Jansa SA (2012). Snake-venom resistance as a mammalian trophic adaptation: lessons from didelphid marsupials. Biological Reviews.

[ref-111] Wasko DK, Sasa M (2010). Habitat selection of the terciopelo (Serpentes: Viperidae: *Bothrops asper*) in a lowland rainforest in Costa Rica. Herpetologica.

[ref-112] Wasko DK, Sasa M (2012). Food resources influence spatial ecology, habitat selection, and foraging behavior in an ambush-hunting snake (Viperidae: *Bothrops asper*): an experimental study. Zoology.

[ref-113] Zehr DR (1962). Stages in the normal development of the common garter snake, T*hamnophis sirtalis sirtalis*. Copeia.

[ref-114] Zipkin EF, DiRenzo GV, Ray JM, Rossman S, Lips KR (2020). Tropical snake diversity collapses after widespread amphibian loss. Science.

