# Supplementary figures and images for: Feeding ecology of the Terciopelo pit viper snake (Bothrops asper) in Ecuador
Source: PeerJ. 2023 Feb 8;11:e14817. doi: 10.7717/peerj.14817 (PMC9921990; doi:10.7717/peerj.14817)

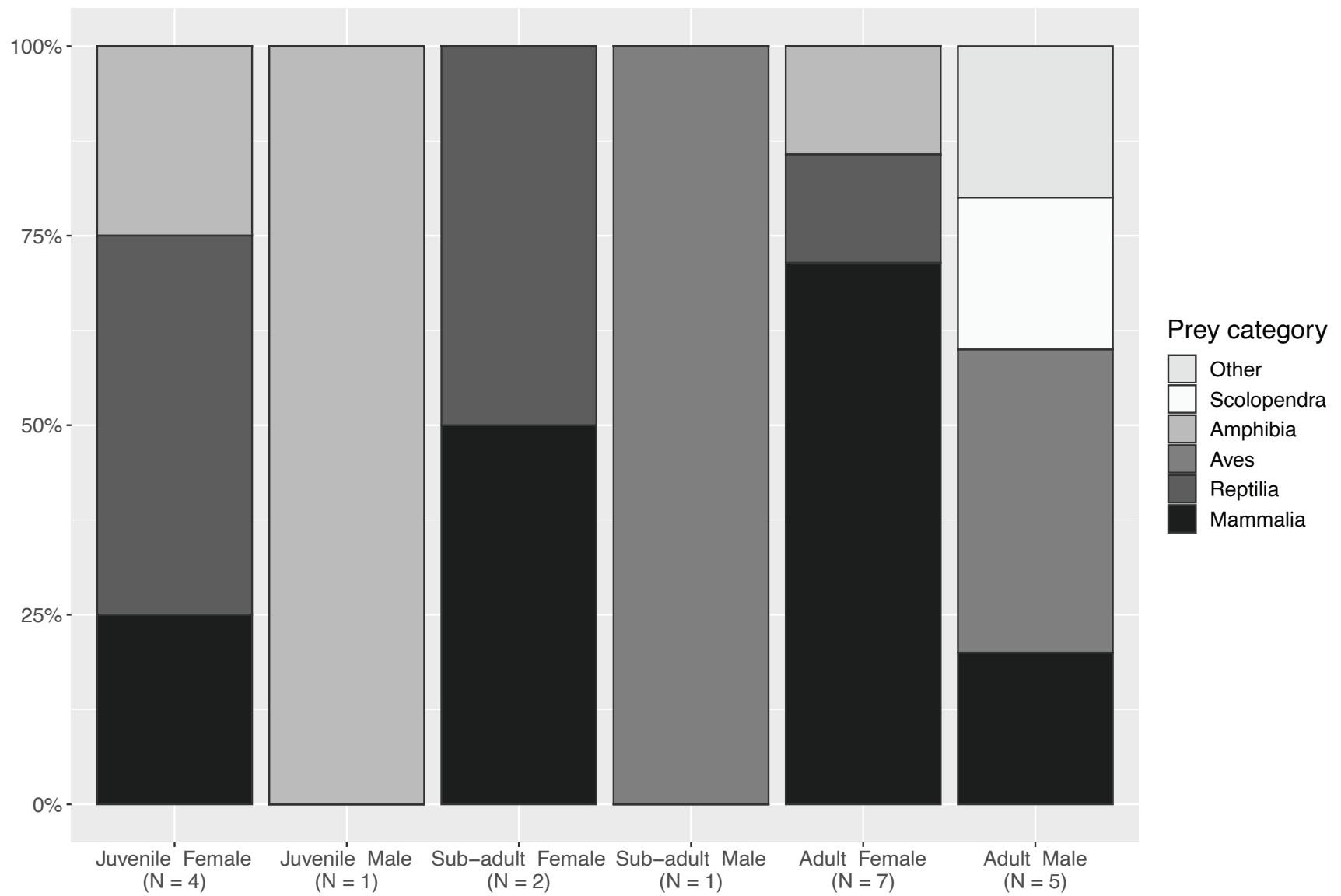

Supplement: Supplemental Information 1 [file peerj-11-14817-s001.pdf]

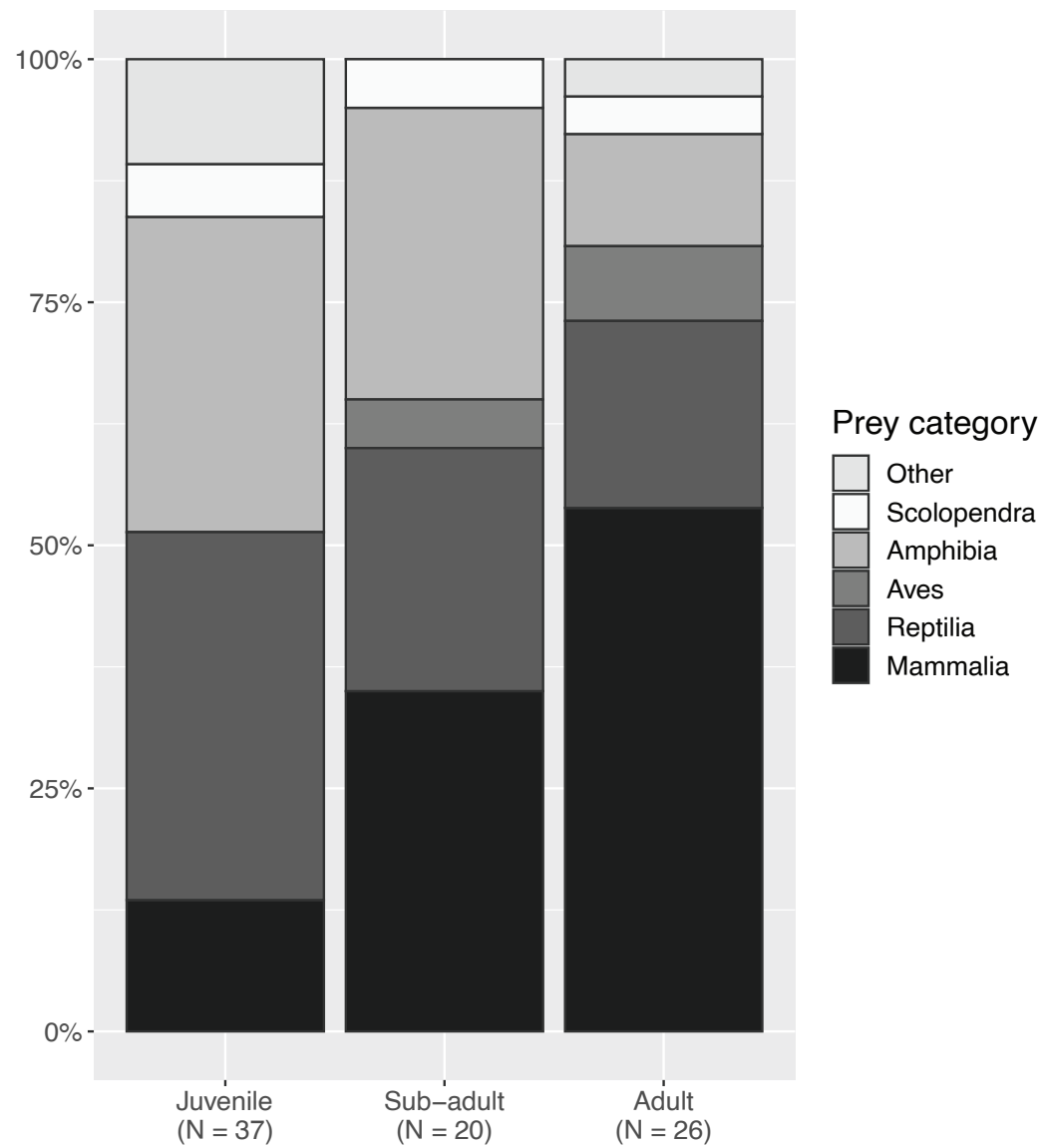

Supplement: Supplemental Information 2 [file peerj-11-14817-s002.pdf]

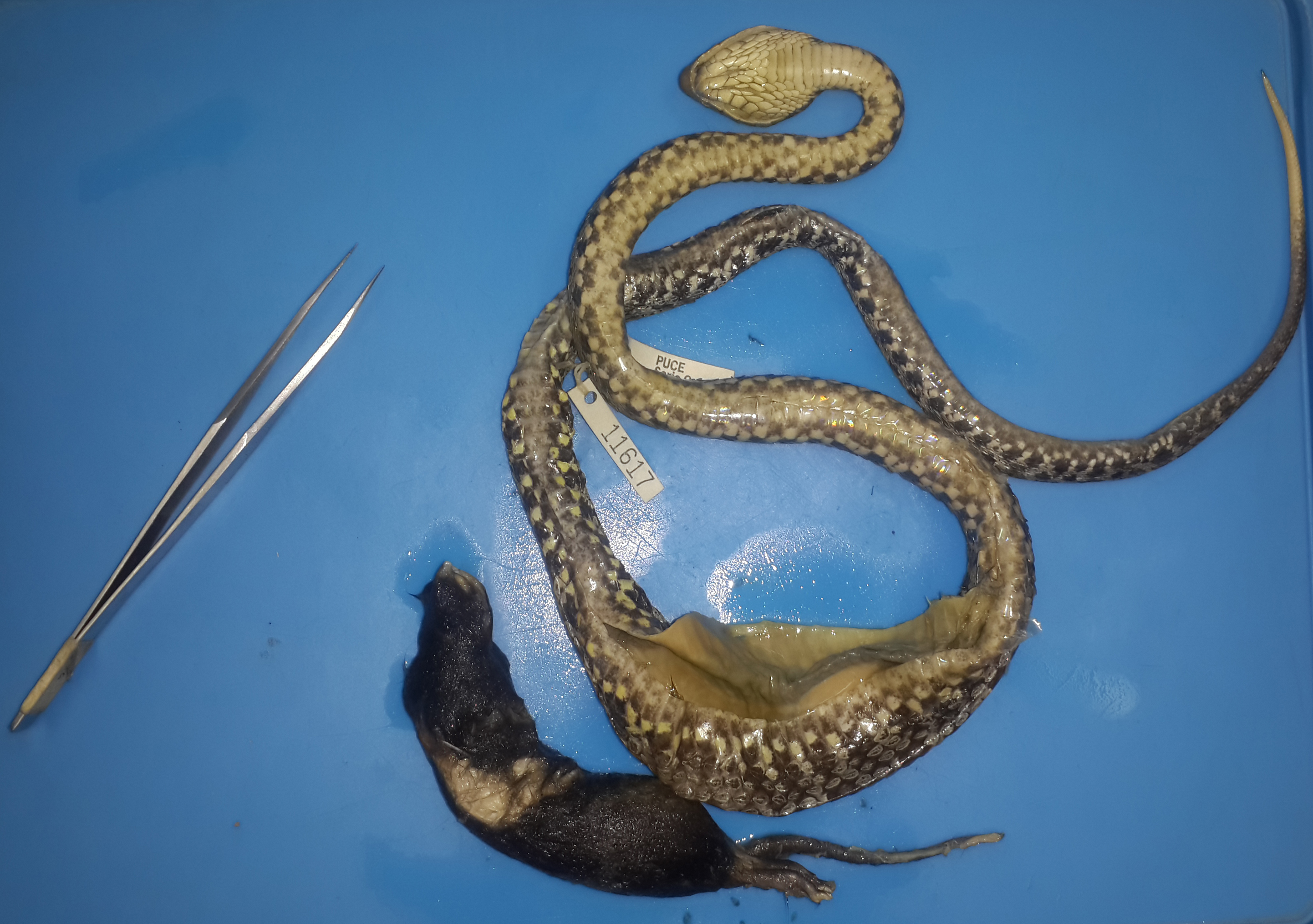

Supplement: Supplemental Information 3 [file peerj-11-14817-s003.jpeg]

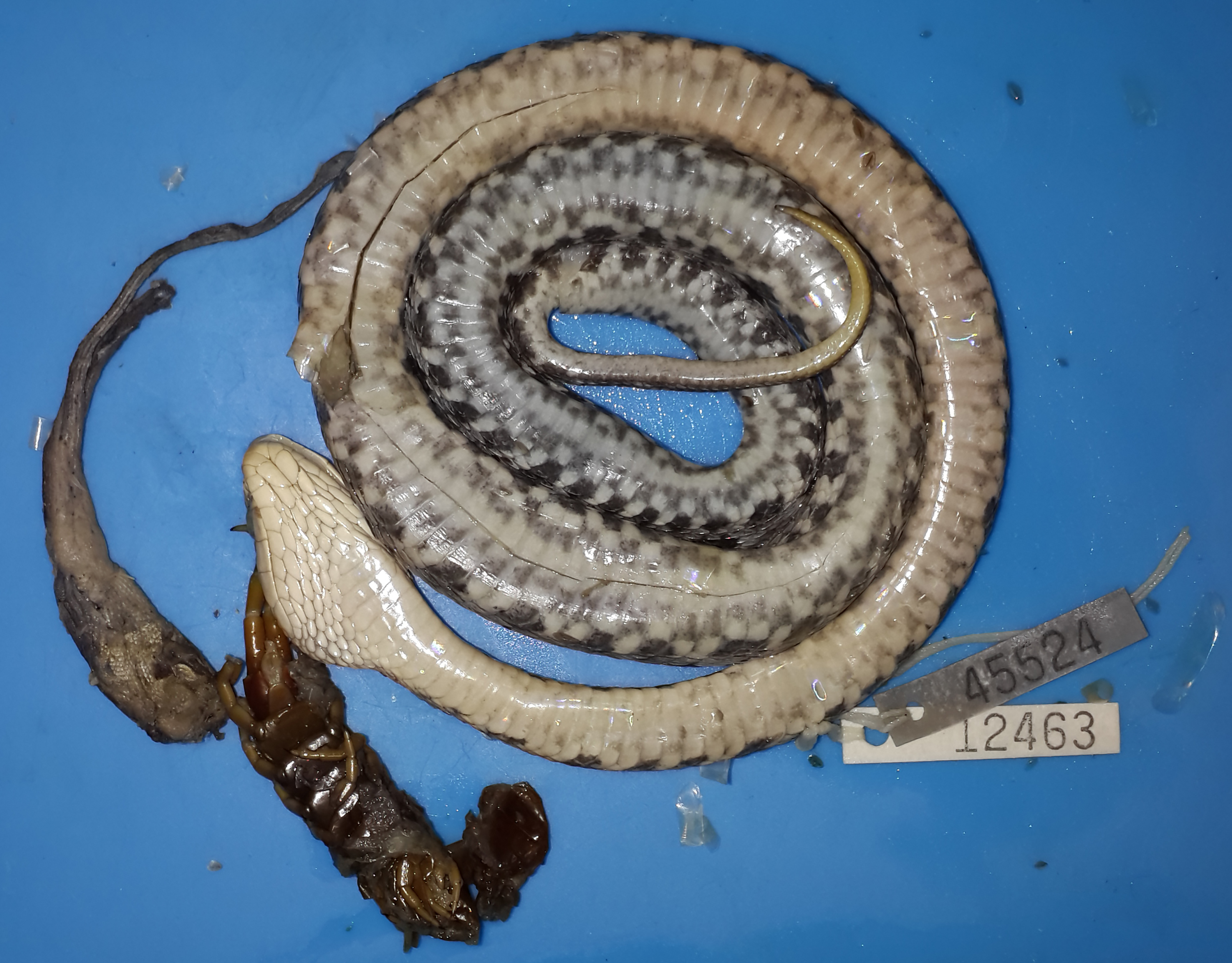

Supplement: Supplemental Information 4 [file peerj-11-14817-s004.jpeg]

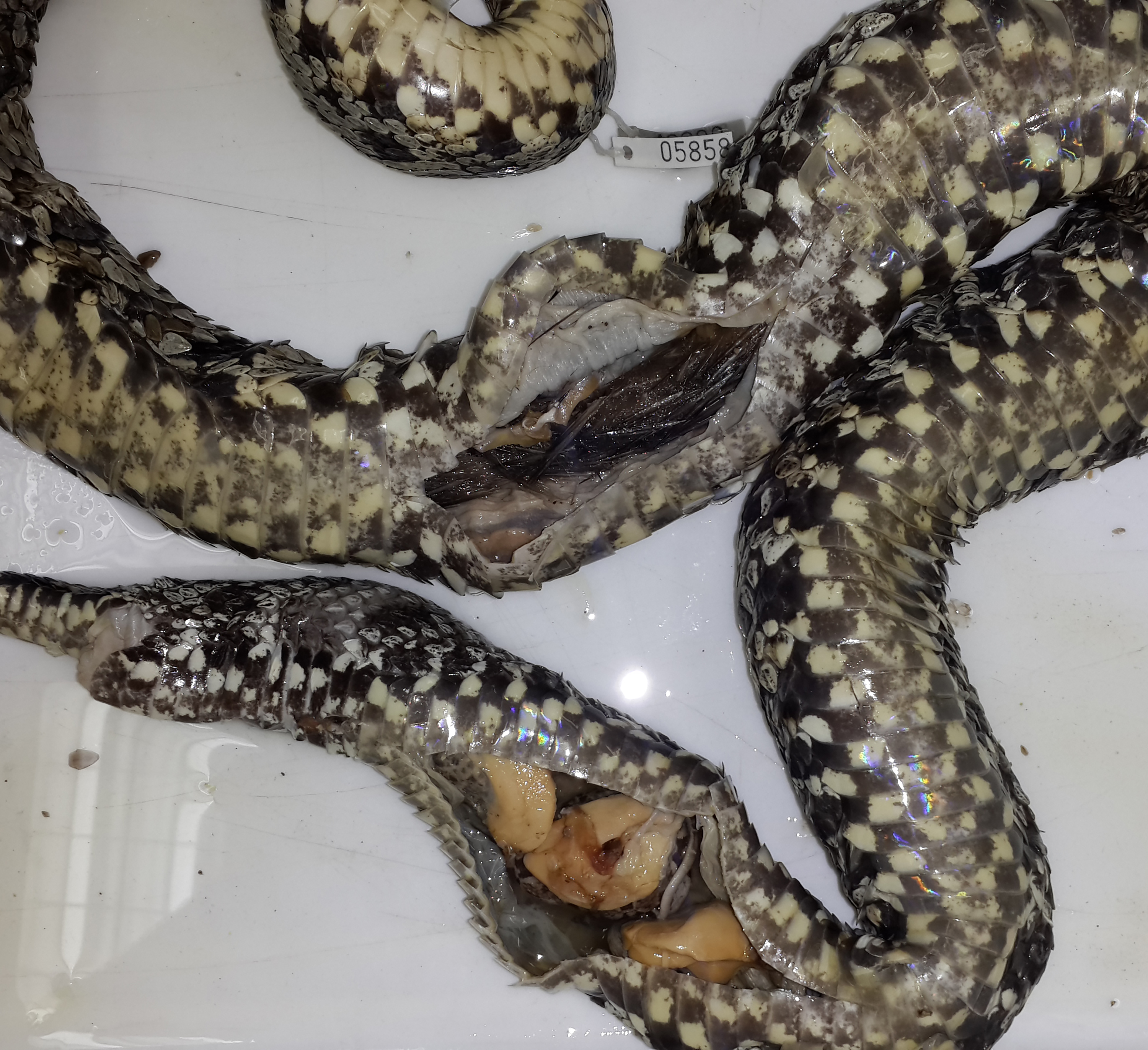

Supplement: Supplemental Information 5 [file peerj-11-14817-s005.jpeg]

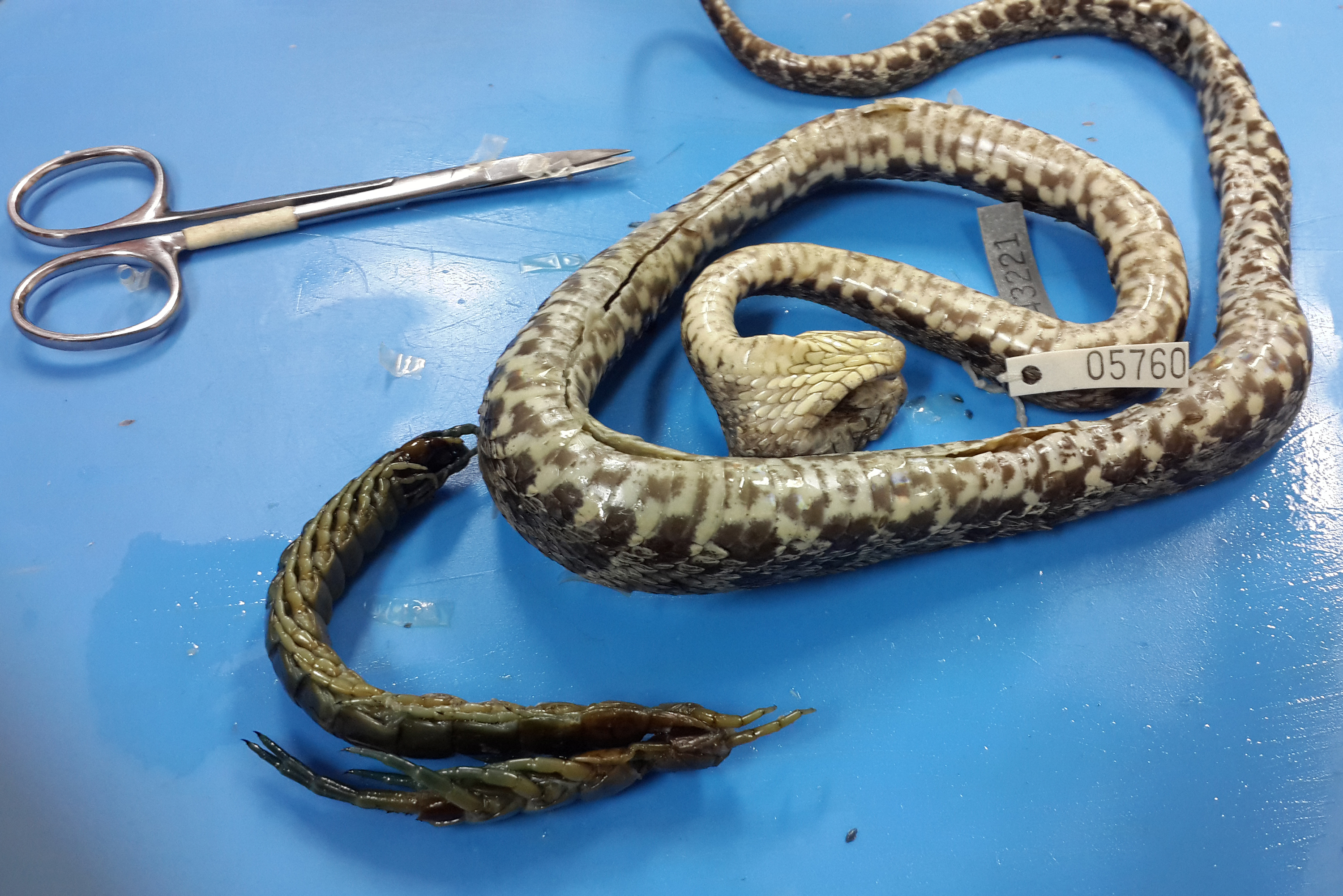

Supplement: Supplemental Information 6 [file peerj-11-14817-s006.jpeg]

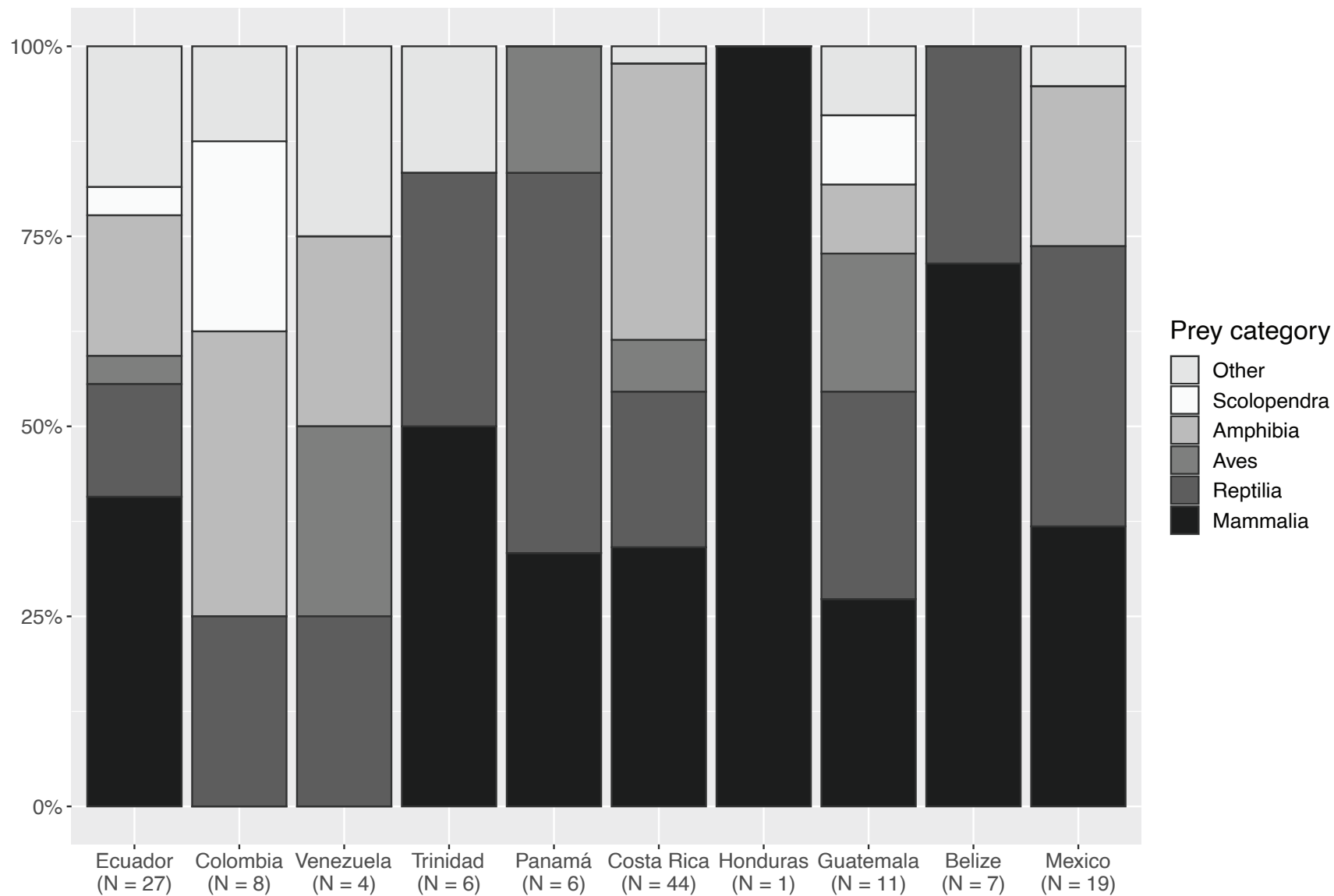

Supplement: Supplemental Information 7 [file peerj-11-14817-s007.pdf]
