# Supplementary material for: Feeding ecology of the Terciopelo pit viper snake (Bothrops asper) in Ecuador
Source: PeerJ. 2023 Feb 8;11:e14817. doi: 10.7717/peerj.14817 (PMC9921990; doi:10.7717/peerj.14817)
Supplement: Supplemental Information 8 [file peerj-11-14817-s008.docx]

**Supplementary Table 1.** Compilation of literature publications with records of diet of *Bothrops asper* throughout its distribution.

| **Author** | **Title** | **Year** | **Language** | **Publication** | **Country** |
| --- | --- | --- | --- | --- | --- |
| Mole RR | The Trinidad snakes | 1924 | English | Journal article | Trinidad |
| Parker HW | The reptiles and batrachians of Gorgona Island, Colombia | 1926 | English | Journal article | Colombia |
| March DDH | Field notes on Barba amarilla (*Bothrops atrox*) | 1928 | English | Short note | Honduras |
| Barbour T, Loveridge A | On *Bothrops atrox* (Linné) | 1929 | English | Short note | Guatemala |
| Nicéforo-María H | Los reptiles y batracios de Honda (Tolima) en el Museo de La Salle | 1930 | Spanish | Journal article | Colombia |
| Picado C | Serpientes venenosas de Costa Rica | 1931 | Spanish | Book | Costa Rica |
| Smith HM | Notes on Mexican amphibians and reptiles | 1947 | English | Short note | Mexico |
| Stuart LC | The amphibians and reptiles of Alta Verapaz, Guatemala | 1948 | English | Book | Guatemala |
| Hirth HF | Observations on the Fer-de -Lance, *Bothrops atrox*, in coastal Costa Rica | 1964 | English | Short note | Costa Rica |
| Sexton OJ, Heatwole H | Life history notes on some Panamanian snakes | 1965 | English | Short note | Panamá |
| Villa B, Lopez-Forment W | Cinco casos de depredacion de pequenos vertebrados en murcielagos de Mexico | 1966 | Spanish | Journal article | Mexico |
| Emsley M | Snakes, and Trinidad and Tobago | 1977 | English | Journal article | Trinidad |
| Henderson RW, Hoevers LG | The seasonal incidence of snakes at a locality in Northern Belize | 1977 | English | Journal article | Belize |
| Solórzano A, Cerdas L | Reproductive biology and distribution of the Terciopelo, *Bothrops asper* Garman (Serpentes: Viperidae), in Costa Rica | 1989 | English | Journal article | Costa Rica |
| Greene HW, Hardy DL | Natural death associated with skeletal injury in the Terciopelo, *Bothrops asper* (Viperidae) | 1989 | English | Short note | Costa Rica |
| Greene HW | The ecological and behavioral context for pitviper evolution | 1992 | English | Book | Colombia |
| Buttenhoff PA, Vogt RC | *Bothrops asper* (Nayuaca) Cannibalism | 1995 | English | Short note | Mexico |
| Buttenhoff PA, Vogt RC | Historia natural de especies (*Bothrops asper*). In: González-Soriano E, Dirzo R, Vogt RC eds., Historia Natural de Región de Los Tuxtlas. Distrito Federal, México: Universidad Nacional Autónoma de México | 1997 | Spanish | Book section | Mexico |
| Greene HW | Snakes: the evolution of mystery in nature | 1997 | English | Book | Costa Rica |
| Murphy JC | Amphibians and reptiles of Trinidad and Tobago | 1997 | English | Book | Trinidad |
| Campbell JA | Amphibians and reptiles of Northern Guatemala, the Yucatán, and Belize | 1999 | English | Book | Guatemala |
| Campbell JA, Lamar WW | The venomous reptiles of the western hemisphere (Vol. 1, No. 2) | 2004 | English | Book | Guatemala |
| Kuch U, Boada C, García F, Torres J, Freire A | *Bothrops asper* (Terciopelo or equis). Diet | 2004 | English | Short note | Ecuador |
| Solórzano A | Serpientes de Costa Rica: distribución, taxonomía e historia natural | 2004 | Spanish | Book | Costa Rica |
| Boada C, Salazar-Valenzuela D, Lascano A, Kuch U | The diet of *Bothrops asper* (Garman, 1884) in the Pacific lowlands of Ecuador | 2005 | English | Short note | Ecuador |
| Cisneros-Heredia DF | Distribution and ecology of the western Ecuador frog *Leptodactylus labrosus* (Amphibia: Anura: Leptodactylidae) | 2006 | English | Journal article | Ecuador |
| Logan CJ, Montero C | *Bothrops asper* (Terciopelo) scavenging behavior | 2009 | English | Short note | Costa Rica |
| Sasa M, Wasko DK, Lamar WW | Natural history of the Terciopelo *Bothrops asper* (Serpentes: Viperidae) in Costa Rica | 2009 | English | Journal article | Costa Rica |
| Hertz A, Natera M, Lotzkat S, Sunyer J,  Mora D | *Bothrops asper* (Mapanare, Lancehead). Prey | 2009 | English | Short note | Venezuela |
| Urbina-Cardona JN | *Bothrops asper* (Terciopelo). Diet | 2009 | English | Short note | Mexico |
| Ryan MJ, Blea NJ, Latella IM, Kull MA | *Leptodactylus savagei* (Smoky Jungle Frog) Antipredator defense | 2010 | English | Short note | Costa Rica |
| Roldan JS, Lucero MF | A prey item not previously recorded for *Bothrops asper*: a case of ophiophagy involving two sympatric pit viper species | 2011 | English | Short note | Colombia |
| Lemos-Espinal JA, Dixon JR | Amphibians and reptiles of San Luis Potosí | 2013 | English | Book | Mexico |
| Voss RS | Opossums (Mammalia: Didelphidae) in the diets of Neotropical pitvipers (Serpentes: Crotalinae): Evidence for alternative coevolutionary outcomes? | 2013 | English | Journal article | Costa Rica |
| Jones M, Straka JR, Kayano K | *Bothrops asper* (Fer-de-Lance). Diet | 2014 | English | Short note | Costa Rica |
| Segovia-Núñez G, de Osma A, Ramírez-Barajas P | *Bothrops asper* (Terciopelo). Diet | 2014 | English | Short note | Ecuador |
| Moody EK | *Bothrops asper* (Fer-de-Lance). Diet and feeding behavior | 2015 | English | Short note | Panamá |
| Rojas Murcia LE, Carvajal Cogollo JE, Cabrejo Bello JA | Reptiles from the seasonal dry  forest the Caribbean region: distribution of habitat and use of food resource | 2016 | Spanish | Journal article | Colombia |
| Valencia JH, Garzón-Tello K, Barragán-Paladines ME, Oxford P | Serpientes venenosas del Ecuador: Sistemática, taxonomía, historia natural, conservación, envenenamiento y aspectos antropológicos | 2016 | Spanish | Book | Ecuador |
| Platt SG, Raineater TR, Meerman JC, Miller SM | Notes on the diet, foraging behavior, and venom of some snakes in Belize | 2016 | English | Note | Belize |
| Cadena-Ortiz H, Barahona A, Bahamonde-Vinueza D, Brito J | Anecdotal predation events of some snakes in Ecuador | 2017 | English | Note | Ecuador |
| Farr WL, Lazcano D | Distribution of *Bothrops asper* in Tamaulipas, Mexico and a review of prey items | 2017 | English | Note | Mexico |
| Sosa-Bartuano A, Añino Ramos Y, Santos A | *Bothrops asper* (Garman, 1883). Diet | 2017 | English | Note | Panamá |
| Díaz-Ricaurte JC | First record of attempted piscivory by *Bothrops asper* (Garman, 1883) (Squamata, Viperidae) on a swamp eel, genus *Synbranchus* | 2018 | English | Note | Colombia |
| Orellana-Vásquez H, Díaz L | Reporte de *Melanomys caliginosus* (Rodentia: Cricetidae) en la dieta de *Bothrops asper* (Squamata: Viperidae) en las estribaciones noroccidentales de los Andes, Chitoa, Santo Domingo de los Tsáchilas, Ecuador | 2019 | Spanish | Note | Ecuador |
| Carbajal Márquez RA, García Balderas CM, Ramírez Valverde T, Cedeño Vázquez JR, Blanco Campos NG | New prey items in the diet of snakes from the Yucatán Peninsula, Mexico | 2019 | English | Note | Mexico |
| Londoño-Quiceno C, Escobar-Lasso S, Zuluaga-Isaza JC, Caicedo-Martínez LS | Predation on Colombian endemic frog *Rheobates palmatus* (Werner, 1899) (Anura: Aromobatidae) by the terciopelo viper *Bothrops asper* (Garman, 1884) (Squamata: Viperidae) | 2020 | English | Note | Colombia |
| Szczygiel H, Page R | When the hunter becomes the hunted: foraging bat attacked by pit viper at frog chorus | 2020 | English | Journal article | Colombia |
| Arteaga A | Fer-de-Lance (*Bothrops asper*). Reptiles of Ecuador | 2020 | English | Book section | Ecuador |
| Gabrysova B, Aznar González de Rueda J, Barrio-Amorós CL | *Bothrops asper* (Terciopelo). Diet/ophiophagy | 2020 | English | Note | Ecuador and Costa Rica |
| Vela RA, Juárez JLC, Calvario ÁIC | Predation on Rainbow Ameivas, *Holcosus undulatus* (sensu lato), and a second record of predation on *H. amphigrammus* (Smith and Laufe 1945) by a Terciopelo (*Bothrops asper*) in Veracruz, Mexico | 2020 | English | Note | Mexico |
| Mata-Lorenzen J, Solórzano A | *Bothrops asper* (Fer-de-lance) diet | 2021 | English | Note | Costa Rica |
| Fujishima K, Fukuyama R, Ishiba Y | *Bothrops asper* (Fer-de-Lance). Diet and ophiophagy | 2021 | English | Note | Costa Rica |
| Fortier R | *Bothrops asper* (Fer-de-lance). Diet | 2021 | English | Note | Panamá |

REFERENCES

**Arteaga A. 2021.** Fer-de-Lance (*Bothrops asper*). Reptiles of Ecuador. Available at <https://www>.reptilesofecuador.com/bothrops_asper.html.

**Barbour T, Loveridge A. 1929.** On *Bothrops atrox* (Linné). *Bulletin of the Antivenin Institute of America* **2**:108 DOI 10.1016/j.toxcx.2020.100037.

**Boada C, Salazar-Valenzuela D, Lascano A, Kuch U. 2005.** The diet of *Bothrops asper* (Garman, 1884) in the Pacific lowlands of Ecuador. *Herpetozoa* **18**:77–79.

**Buttenhoff PA, Vogt RC. 1995.** *Bothrops asper* (Nayuaca). Cannibalism. *Herpetological Review* **26**:146–147.

**Buttenhoff PA, Vogt RC. 1997.** Historia natural de especies (*Bothrops asper*). In: González-Soriano E, Dirzo R, Vogt RC, eds. *Historia Natural de Región de Los Tuxtlas*. Distrito Federal, México: Universidad Nacional Autónoma de México, 478–480.

**Cadena-Ortiz H, Barahona A, Bahamonde-Vinueza D, Brito J. 2017.** Anecdotal predation events of some snakes in Ecuador. *Herpetozoa* **30**:93–96.

**Campbell JA. 1999.** Amphibians and reptiles of northern Guatemala, the Yucatán, and Belize. Vol. 4. Norman, Oklahoma: University of Oklahoma Press.

**Campbell JA, Lamar WW. 2004.** *The venomous reptiles of the western hemisphere*. Vol. 1. Ithaca, NY: Comstock Publishing Associates.

**Carbajal-Márquez RA, García-Balderas CM, Ramírez-Valverde T, Cedeño-Vázquez R, Blanco-Campos NG. 2019.** New prey items in the diet of snakes from the Yucatán Peninsula, Mexico. *Cuadernos de Herpetología* **33(2)**:71–74 DOI 10.31017/CdH.2019.

**Cisneros-Heredia DF. 2006.** Distribution and ecology of the western Ecuador frog *Leptodactylus labrosus* (Amphibia: Anura: Leptodactylidae). *Zoological Research* **27**:225–234.

**Díaz-Ricaurte JC. 2018.** First record of attempted piscivory by *Bothrops asper* (Garman, 1883) (Squamata, Viperidae) on a swamp eel, genus *Synbranchus*. *Herpetology Notes* **11:**835–837.

**Emsley M. 1977.** Snakes, and Trinidad and Tobago. *Maryland Herpetological Society Bulletin* **13**:201–304.

**Farr WL, Lazcano D. 2017.** Distribution of *Bothrops asper* in Tamaulipas, Mexico and a review of prey items. *The Southwestern Naturalist* **62(1)**:77–84 DOI 10.1894/0038-4909-62.1.77.

**Fortier R. 2021.** *Bothrops asper* (Fer-de-Lance). Diet. *Herpetological Review* **52:**148.

**Fujishima K, Fukuyama R, Ishiba Y. 2021.** *Bothrops asper* (Fer-de-Lance). Diet and ophiophagy. *Herpetological Review* **52:**658.

**Gabrysova B, Aznar Gonzáles de Rueda J, Barrio-Amorós CL. 2020.** *Bothrops asper* (Terciopelo). Diet/Ophiophagy. *Herpetological Review* **51**:859–860.

**Greene HW. 1992.** The ecological and behavioral context for pitviper evolution. In: Greene HW, Campbell JA, Brodie ED, eds. *Biology of the Pitvipers.* Tyler, Texas: Selva Press, 107–117.

**Greene HW. 1997.** *Snakes: the evolution of mystery in nature.* Los Angeles, USA: University of California Press.

**Greene HW, Hardy DL. 1989.** Natural death associated with skeletal injury in the terciopelo, *Bothrops asper* (Viperidae). *Copeia* **1989(4)**:1036 DOI 10.2307/1445992.

**Henderson R, Hoevers L. 1977.** The seasonal incidence of snakes at a locality in Northern Belize. *Copeia* **1977(2)**:349 DOI 10.2307/1443914.

**Hertz A, Natera M, Lotzkat S, Sunyer J, Mora D. 2009.** *Bothrops asper* (Mapanare, Lancehead). Prey. *Herpetological Review* **40**:230.

**Hirth HF. 1964.** Observations on the Fer-de-Lance, *Bothrops atrox*, in coastal Costa Rica. *Copeia* **1964(2)**:453 DOI 10.2307/1441044.

**Jones MA, Straka JR, Kayano K. 2014.** *Bothrops asper* (Fer-de-Lance). Diet. *Herpetological Review* **45**:3.

**Kuch U, Boada C, García F, Torres J, Freire A. 2004.** *Bothrops asper* (Terciopelo or equis). Diet. *Herpetological Review* **35**:273–274.

**Lemos-Espinal JA, Dixon JR. 2013.** *Amphibians and reptiles of San Luis Potosí.* Eagle Mountain, Utah: Eagle Mountain Publishing.

**Logan CJ, Montero C. 2009.** *Bothrops asper* (Terciopelo) scavenging behavior. *Herpetological Review* **40:**352–352 DOI 10.17863/CAM.5932.

**Londoño-Quiceno C, Escobar-Lasso S, Zuluaga-Isaza JC, Caicedo-Martínez LS. 2020.** Predation on Colombian endemic frog *Rheobates palmatus* (Werner, 1899) (Anura: Aromobatidae) by the Terciopelo viper *Bothrops asper* (Garman, 1884) (Squamata: Viperidae). *Herpetology Notes* **13:**641–644.

**March DDH.** 1928. Field notes on Barba amarilla (*Bothrops atrox*). *Bulletin of the Antivenin Institute of America* **1**:92–97.

**Mata-Lorenzen J, Solórzano A. 2021.** *Bothrops asper* (Fer-de-lance) diet. *Herpetological Review* **52**:148–149.

**Mole RR. 1924.** The Trinidad snakes. *Proceedings of the Zoological Society of London* **94(1)**:235–278 DOI 10.1111/j.1096-3642.1924.tb01500.x.

**Moody EK. 2015.** *Bothrops asper* (Fer-de-Lance). Diet and feeding behavior. *Herpetological Review* **46:**266–267.

**Murphy JC. 1997.** *Amphibians and reptiles of Trinidad and Tobago.* Malabar, Florida: Krieger.

**Nicéforo-María H. 1930.** Los reptiles y batracios de Honda (Tolima) en el Museo de La Salle. *Revista Sociedad Colombiana de Ciencias Naturales* **19**:96–104.

**Orellana-Vásquez H, Díaz L. 2019.** Reporte de *Melanomys caliginosus* (Rodentia: Cricetidae) en la dieta de *Bothrops asper* (Squamata: Viperidae) en las estribaciones noroccidentales de los Andes, Chitoa, Santo Domingo de los Tsáchilas, Ecuador. *ACI Avances en Ciencias e Ingenierías* **11(2)**:266–273 DOI 10.18272/aci.v11i2.1064.

**Parker HW. 1926.** The reptiles and batrachians of Gorgona Island, Colombia. *Annals and Magazine of Natural History* **17**(101):549–554 DOI 10.1080/00222932608633442.

**Picado C. 1931.** *Serpientes venenosas de Costa Rica.* San José, Costa Rica: Imprenta Alsina. Sauter, Arias & Co.

**Platt SG, Rainwater TR, Meerman JC, Miller SM. 2016.** Notes on the diet, foraging behavior, and venom of some snakes in Belize. *Mesoamerican Herpetology* **1**:162–170.

**Rojas Murcia LE, Carvajal Cogollo JE, Cabrejo Bello JA. 2016.** Reptiles from the seasonal dry forest the Caribbean region: distribution of habitat and use of food resource. *Acta Biologica Colombiana* **21:**365–377 DOI 10.15446/abc.v21n2.49393.

**Roldan JSM, Lucero MF. 2011.** A prey item not previously recorded for *Bothrops asper*: a case of ophiophagy involving two sympatric pit viper species. *Herpetotropicos: Tropical Amphibians & Reptiles* **5(2)**:107–109.

**Ryan M, Blea N, Latella I, Kull M. 2010.** *Leptodactylus savagei* (smoky jungle frog). Antipredator defense. *Herpetological Review* **41**:337–338.

**Sasa M, Wasko DK, Lamar WW. 2009.** Natural history of the terciopelo *Bothrops asper* (Serpentes: Viperidae) in Costa Rica. *Toxicon* **54(7)**:904–922 DOI 10.1016/j.toxicon.2009.06.024.

**Segovia-Núñez G, de Osma A, Ramírez-Barajas P. 2014.** *Bothrops asper* (Terciopelo). Diet. *Herpetological Review* **45**:512–513.

**Sexton OJ, Heatwole H. 1965.** Life history notes on some Panamanian snakes*. Caribbean Journal of Science* **5:**39–43.

**Smith HM. 1947.** Notes on Mexican amphibians and reptiles. *Journal of the Washington Academy of Sciences* **37**:408–412.

**Solórzano A. 2004.** *Serpientes de Costa Rica: distribución, taxonomía e historia natural.* Costa Rica: Editorial INBio.

**Solórzano A, Cerdas L. 1989.** Reproductive biology and distribution of the terciopelo, *Bothrops asper* Garman (Serpentes: Viperidae), in Costa Rica. *Herpetologica* **45**:444–450.

**Sosa-Bartuano A, Añino Ramos Y, Santos A. 2017.** *Bothrops asper* (Garman, 1883). Diet. *Mesoamerican Herpetology* **4**:423–424.

**Stuart LC. 1948.** *The amphibians and reptiles of Alta Verapaz, Guatemala.* Ann Arbor: University of Michigan Press.

**Szczygieł H, Page R. 2020.** When the hunter becomes the hunted: foraging bat attacked by pit viper at frog chorus. *Ecology* **101(10)**:e03111 DOI 10.1002/ecy.3111.

**Urbina-Cardona JN. 2009.** *Bothrops asper* (Terciopelo). Diet. *Herpetological Review* **40:**94.

**Valencia JH, Garzón-Tello K, Barragán-Paladines ME, Oxford P. 2016.** *Serpientes venenosas del Ecuador: sistemática, taxonomía, historia natural, conservación, envenenamiento y aspectos antropológicos.* Quito, Ecuador: Fundación Herpetológica Gustavo Orcés.

**Vela RA, Juárez JLC, Calvario ÁIC. 2020.** Predation on Rainbow Ameivas, *Holcosus undulatus* (sensu lato), and a second record of predation on *H. amphigrammus* (Smith and Laufe 1945) by a terciopelo (*Bothrops asper*) in Veracruz, Mexico. *Reptiles & Amphibians* **27**:422–425 DOI 10.17161/randa.v27i3.14860.

**Villa B, Lopez-Forment W. 1966.** Cinco casos de depredación de pequeños vertebrados en murciélagos de Mexico. *Anales del Instituto de Biología de la Universidad de Mexico* **37**:187–193.

**Voss RS. 2013.** Opossums (Mammalia: Didelphidae) in the diets of Neotropical pitvipers (Serpentes: Crotalinae): evidence for alternative coevolutionary outcomes? *Toxicon* **66(6)**:1–6 DOI 10.1016/j.toxicon.2013.01.013.
