# Supplementary material for: Feeding ecology of the Terciopelo pit viper snake (Bothrops asper) in Ecuador
Source: PeerJ. 2023 Feb 8;11:e14817. doi: 10.7717/peerj.14817 (PMC9921990; doi:10.7717/peerj.14817)
Supplement: Supplemental Information 9 [file peerj-11-14817-s009.docx]

**Supplementary Table 2.** Prey taxa reported from the diet of *Bothrops asper* throughout its distribution.

| Taxa |  | Country | Reference | Year |  |
| --- | --- | --- | --- | --- | --- |
| Invertebrates |  |  |  |  |  |
|  |  | Mexico | Buttenhoff & Vogt | 1997 | ^3^ |
| **Malacostraca** |  |  |  |  |  |
|  | *“Crayfish”* | Trinidad | Emsley | 1977 |  |
| **Chilopoda** |  |  |  |  |  |
| Scolopendromorpha |  | Colombia | Greene | 1992 |  |
|  |  | Guatemala | Campbell | 1999 |  |
|  |  | Ecuador | Boada et al. | 2005 |  |
|  |  | Ecuador | This study |  |  |
|  | *Scolopendra angulata* | Colombia | Parker | 1926 |  |
| **Insecta** |  |  |  |  |  |
| Coleoptera |  | Ecuador | Boada et al. | 2005 |  |
| Diptera |  | Ecuador | Boada et al. | 2005 |  |
| Hemiptera |  | Ecuador | Boada et al. | 2005 |  |
| Hymenoptera |  | Ecuador | Boada et al. | 2005 |  |
| Orthoptera |  | Costa Rica | Sasa, Wasko & Lamar | 2009 |  |
|  |  | Ecuador | Boada et al. | 2005 |  |
|  |  | Guatemala | Campbell | 1999 |  |
| **Actinopterygii** |  |  |  |  |  |
| Synbranchidae | *Synbranchus* sp. | Colombia | Díaz-Ricaurte | 2018 |  |
|  | *Synbranchus marmoratus* | Venezuela | Hertz et al. | 2009 |  |
| **Amphibia** |  |  |  |  |  |
| Anura |  | Costa Rica | Solórzano & Cerdas | 1989 |  |
|  |  | Costa Rica | Logan & Montero | 2009 |  |
|  |  | Ecuador | Boada et al. | 2005 |  |
|  |  | Ecuador | This study |  |  |
| Aromobatidae | *Rheobates palmatus* | Colombia | Londoño-Quiceno et al. | 2020 |  |
| Bufonidae | *Rhinella marina* | Costa Rica | Sasa, Wasko & Lamar | 2009 |  |
|  |  | Ecuador | Boada et al. | 2005 |  |
| Craugastoridae | *Craugastor fitzingeri* | Costa Rica | Sasa, Wasko & Lamar | 2009 |  |
|  | *Craugastor rhodopis* | Mexico | Buttenhoff & Vogt | 1997 | ^3^ |
|  | *Pristimantis achatinus* | Ecuador | Boada et al. | 2005 |  |
|  |  | Ecuador | This study |  |  |
|  | *Pristimantis* sp. | Ecuador | This study |  |  |
| Dendrobatidae |  | Venezuela | Lemos-Espinal & Dixon | 2013 | ^3^ |
| Eleutherodactylidae | *Eleutherodactylus* sp. | Costa Rica | Greene | 1997 |  |
|  |  | Guatemala | Campbell | 1999 |  |
| Gymnophiona | *Caecilia* cf. *caribea* | Colombia | Rojas Murcia, Carvajal Cogollo & Cabrejo Bello | 2016 |  |
|  | *Dermophis parviceps* | Costa Rica | Jones, Straka & Kayano | 2014 |  |
|  | *Gymnopis multiplicata* | Costa Rica | Mata-Lorenzen & Solórzano | 2021 |  |
| Hylidae | *Smilisca* sp. | Mexico | Smith | 1947 |  |
|  | *Smilisca baudinii* | Mexico | Buttenhoff & Vogt | 1997 | ^3^ |
|  | *Smilisca phaeota* | Ecuador | Valencia et al. | 2016 |  |
|  |  | Costa Rica | Sasa, Wasko & Lamar | 2009 |  |
|  | *Smilisca sordida* | Costa Rica | Sasa, Wasko & Lamar | 2009 |  |
| Leptodactylidae | *Leptodactylus* sp. | Colombia | Rojas Murcia, Carvajal Cogollo & Cabrejo Bello | 2016 |  |
|  | *Leptodactylus bolivianus* | Costa Rica | Sasa, Wasko & Lamar | 2009 |  |
|  | *Leptodactylus labialis* | Mexico | Smith | 1947 |  |
|  | *Leptodactylus labrosus* | Ecuador | Cisneros-Heredia | 2006 |  |
|  | *Leptodactylus savagei* | Costa Rica | Sasa, Wasko & Lamar | 2009 |  |
|  |  | Costa Rica | Ryan et al., 2010 | 2010 |  |
|  | *Leptodactylus ventrimaculatus* | Ecuador | This study |  |  |
| Ranidae | *Lithobates forreri* | Costa Rica | Sasa, Wasko & Lamar | 2009 |  |
|  |  | Costa Rica | Campbell & Lamar | 2004 |  |
|  | *Lithobates vaillanti* | Costa Rica | Sasa, Wasko & Lamar | 2009 |  |
|  | *Lithobates warszewitschii* | Costa Rica | Sasa, Wasko & Lamar | 2009 |  |
| **Reptilia** |  |  |  |  |  |
| Squamata |  | Ecuador | Buttenhoff & Vogt | 1995 |  |
|  |  | Ecuador | This study |  |  |
|  |  | Ecuador | Boada et al. | 2005 |  |
|  |  | Costa Rica | Solórzano & Cerdas | 1989 |  |
| Amphisbaenidae | *Amphisbaena alba* | Venezuela | Hertz et al. | 2009 |  |
| Alopoglossidae | *Ptychoglossus* sp. | Ecuador | Valencia et al. | 2016 |  |
| Corytophanidae | *Basiliscus basiliscus* | Panamá | Fortier | 2021 |  |
| Dactyloidae | *Anolis* sp. | Guatemala | Campbell | 1999 |  |
|  | *Anolis bitectus* | Ecuador | This study |  |  |
|  | *Anolis limifrons* | Panamá | Sexton & Heatwole | 1965 |  |
| Iguanidae | *Ctenosaura similis* | Costa Rica | Sasa, Wasko & Lamar | 2009 |  |
| Phrynosomatidae | *Sceloporus chrysostictus* | Belize | Platt et al. | 2016 |  |
| Scincidae | *Scincella cherriei* | Mexico | Carbajal-Márquez et al. | 2019 |  |
|  |  | Guatemala | Stuart | 1948 |  |
| Scincidae | *“Skinks”* | Guatemala | Campbell | 1999 |  |
| Sphaerodactylidae | *Gonatodes albogularis* | Colombia | Nicéforo-María | 1930 | ^1,3^ |
| Teiidae | *Holcosus amphigrammus* | Mexico | Vela, Juárez & Calvario | 2020 |  |
|  |  | Mexico | Buttenhoff & Vogt | 1997 | ^3,5^ |
|  | *Holcosus festivus* | Costa Rica | Sasa, Wasko & Lamar | 2009 |  |
|  |  | Panamá | Sosa-Bartuano, Añino Ramos & Santos | 2017 |  |
|  | *Medopheos edracanthus* | Ecuador | This study |  |  |
| Xantusiidae | *Lepidophyma flavimaculatum* | Belize | Platt et al. | 2016 |  |
|  | *Lepidophyma reticulatum* | Costa Rica | Sasa, Wasko & Lamar | 2009 |  |
|  | *Lepidophyma tuxtlae* | Mexico | Urbina-Cardona | 2009 |  |
| Colubridae | *Dipsas andiana* | Ecuador | Arteaga | 2021 |  |
|  |  | Ecuador | Gabrysova, Aznar Gonzáles de Rueda & Barrio-Amorós | 2020 |  |
|  | *Enuliophis sclateri* | Costa Rica | Fujishima, Fukuyama & Ishiba | 2021 |  |
|  | *Erythrolamprus bizona* | Costa Rica | Solórzano | 2004 |  |
|  |  | Costa Rica | Solórzano | 2004 |  |
|  | *Ninia atrata* | Trinidad | Murphy | 1997 | ^3^ |
|  |  | Trinidad | Murphy | 1997 | ^1^ |
|  | *Ninia sebae* | Mexico | Carbajal-Márquez et al. | 2019 |  |
|  |  | Costa Rica | Sasa, Wasko & Lamar | 2009 |  |
|  | *Tantilla supracincta* | Costa Rica | Gabrysova, Aznar Gonzáles de Rueda & Barrio-Amorós | 2020 |  |
| Viperidae | *Bothrops asper* | Mexico | Buttenhoff & Vogt | 1997 | ^3^ |
|  |  | Mexico | Buttenhoff & Vogt | 1995 |  |
|  | *Porthidium lansbergii* | Colombia | Roldan & Lucero | 2011 |  |
| **Aves** |  |  |  |  |  |
|  |  | Costa Rica | Picado | 1931 | ^3^ |
|  |  | Guatemala | Barbour & Loveridge | 1929 | ^1,3^ |
| Passeriformes |  | Costa Rica | Sasa, Wasko & Lamar | 2009 |  |
|  |  | Guatemala | Barbour & Loveridge | 1929 | ^1^ |
| Thraupidae | *Eucometis penicillata* | Panamá | Moody | 2015 |  |
|  | *Volatinia jacarina* | Costa Rica | Sasa, Wasko & Lamar | 2009 |  |
| Troglodytidae | *Thryothorus nigricapillus* | Ecuador | Boada et al. | 2005 |  |
|  | *Troglodytes* sp. | Venezuela | Hertz et al. | 2009 |  |
| **Mammalia** |  |  |  |  |  |
|  |  | Ecuador | This study |  |  |
|  |  | Panamá | Sexton & Heatwole | 1965 |  |
|  |  | Guatemala | Stuart | 1948 |  |
|  |  | Belize | Platt et al. | 2016 |  |
|  |  | Ecuador | Kuch et al. | 2004 |  |
|  |  | Belize | Henderson & Hoevers | 1977 |  |
|  |  | Mexico | Buttenhoff & Vogt | 1995 |  |
| Didelphidae | *“Opossums”* | Honduras | March | 1928 |  |
|  |  | Costa Rica | Picado | 1931 |  |
|  | *Caluromys derbianus* | Costa Rica | Hirth | 1964 |  |
|  | *Caluromys philander* | Trinidad | Mole | 1924 |  |
|  | *Didelphis* sp. | Mexico | Buttenhoff & Vogt | 1997 | ^3^ |
|  | *Didelphis marsupialis* | Costa Rica | Voss | 2013 |  |
|  |  | Costa Rica | Greene & Hardy | 1989 | ^2^ |
|  | *Marmosops* sp. | Ecuador | This study |  |  |
|  | *Philander opossum* | Guatemala | Campbell | 1999 |  |
|  |  | Costa Rica | Voss | 2013 |  |
|  |  | Costa Rica | Sasa, Wasko & Lamar | 2009 |  |
| Rodentia |  | Belize | Platt et al. | 2016 |  |
|  |  | Ecuador | Kuch et al. | 2004 |  |
|  |  | Ecuador | Boada et al. | 2005 |  |
|  |  | Costa Rica | Hirth | 1964 |  |
|  |  | Trinidad | Mole | 1924 |  |
| Chiroptera | *Glossophaga soricina* | Mexico | Villa & Lopez-Forment | 1966 | ^4^ |
|  | *Trachops cirrhosus* | Panamá | Szczygieł & Page | 2020 |  |
| Cricetidae | *Cricetidae* | Ecuador | This study |  |  |
|  | *Handleyomys* sp. | Ecuador | This study |  |  |
|  | *Melanomys* sp. | Ecuador | This study |  |  |
|  | *Melanomys caliginosus* | Costa Rica | Sasa, Wasko & Lamar | 2009 |  |
|  | *Melanomys caliginosus* | Ecuador | Orellana-Vásquez & Díaz | 2019 |  |
|  | *Microryzomys* sp. | Ecuador | This study |  |  |
|  | *Nephelomys moerex* | Ecuador | This study |  |  |
|  | *Oligoryzomys* sp. | Ecuador | This study |  |  |
|  | *Oligoryzomys fulvescens* | Costa Rica | Sasa, Wasko & Lamar | 2009 |  |
|  | *Oryzomys* sp. | Costa Rica | Sasa, Wasko & Lamar | 2009 |  |
|  | *Ototylomys phyllotis* | Belize | Platt et al. | 2016 |  |
|  | *Sigmodon* sp. | Ecuador | This study |  |  |
|  | *Sigmodon hirsutus* | Costa Rica | Sasa, Wasko & Lamar | 2009 |  |
|  | *Sigmodon peruanus* | Ecuador | Kuch et al. | 2004 |  |
|  | *Sigmodontomys* sp. | Ecuador | Valencia et al. | 2016 |  |
|  | *Thomasomys* sp. | Ecuador | This study |  |  |
|  | *Transandinomys* sp. | Ecuador | This study |  |  |
|  | *“Wood Rat” [Neotoma]* | Trinidad | Mole | 1924 |  |
| Dasyproctidae | *Dasyprocta punctata* | Ecuador | Cadena-Ortiz et al. | 2017 |  |
| Echimydae | *Proechimys* sp. | Ecuador | This study |  |  |
|  | *Proechimys semispinosus* | Ecuador | This study |  |  |
|  |  | Costa Rica | Sasa, Wasko & Lamar | 2009 |  |
|  |  | Ecuador | Segovia-Núñez, de Osma & Ramírez-Barajas | 2014 |  |
| Erethizontidae | *Coendou rothschildi* | Ecuador | Segovia-Núñez, de Osma & Ramírez-Barajas | 2014 |  |
| Heteromyidae | *Heteromys* sp. | Guatemala | Campbell | 1999 |  |
|  | *Heteromys desmarestianus* | Costa Rica | Sasa, Wasko & Lamar | 2009 |  |
| Lagomorpha | *Sylvilagus brasiliensis* | Costa Rica | Greene & Hardy | 1989 |  |
| Muridae |  | Ecuador | Kuch et al. | 2004 |  |
|  | *Mus musculus* | Costa Rica | Sasa, Wasko & Lamar | 2009 |  |
|  |  | Mexico | Carbajal-Márquez et al. | 2019 |  |
|  | *Rattus rattus* | Mexico | Buttenhoff & Vogt | 1997 | ^3^ |
|  |  | Ecuador | Valencia et al. | 2016 |  |
|  |  | Mexico | Buttenhoff & Vogt | 1995 |  |
| Soricidae | *Cryptotis sp.* | Belize | Platt et al. | 2016 |  |
|  | *Cryptotis parva* | Mexico | Farr & Lazcano | 2017 |  |
| Cited by: ^1^Sasa, Wasko and Lamar (2009); ^2^Voss (2013); ^3^Farr and Lazcano (2017); ^4^Szczygieł and Page (2020), ^5^Vela, Juárez and Calvario (2020) | | | | |  |

REFERENCES

**Arteaga A. 2021.** Fer-de-Lance (*Bothrops asper*). Reptiles of Ecuador. Available at <https://www>.reptilesofecuador.com/bothrops_asper.html.

**Barbour T, Loveridge A. 1929.** On *Bothrops atrox* (Linné). *Bulletin of the Antivenin Institute of America* **2**:108 DOI 10.1016/j.toxcx.2020.100037.

**Boada C, Salazar-Valenzuela D, Lascano A, Kuch U. 2005.** The diet of *Bothrops asper* (Garman, 1884) in the Pacific lowlands of Ecuador. *Herpetozoa* **18**:77–79.

**Buttenhoff PA, Vogt RC. 1995.** *Bothrops asper* (Nayuaca). Cannibalism. *Herpetological Review* **26**:146–147.

**Buttenhoff PA, Vogt RC. 1997.** Historia natural de especies (*Bothrops asper*). In: González-Soriano E, Dirzo R, Vogt RC, eds. *Historia Natural de Región de Los Tuxtlas*. Distrito Federal, México: Universidad Nacional Autónoma de México, 478–480.

**Cadena-Ortiz H, Barahona A, Bahamonde-Vinueza D, Brito J. 2017.** Anecdotal predation events of some snakes in Ecuador. *Herpetozoa* **30**:93–96.

**Campbell JA. 1999.** Amphibians and reptiles of northern Guatemala, the Yucatán, and Belize. Vol. 4. Norman, Oklahoma: University of Oklahoma Press.

**Campbell JA, Lamar WW. 2004.** *The venomous reptiles of the western hemisphere*. Vol. 1. Ithaca, NY: Comstock Publishing Associates.

**Carbajal-Márquez RA, García-Balderas CM, Ramírez-Valverde T, Cedeño-Vázquez R, Blanco-Campos NG. 2019.** New prey items in the diet of snakes from the Yucatán Peninsula, Mexico. *Cuadernos de Herpetología* **33(2)**:71–74 DOI 10.31017/CdH.2019.

**Cisneros-Heredia DF. 2006.** Distribution and ecology of the western Ecuador frog *Leptodactylus labrosus* (Amphibia: Anura: Leptodactylidae). *Zoological Research* **27**:225–234.

**Díaz-Ricaurte JC. 2018.** First record of attempted piscivory by *Bothrops asper* (Garman, 1883) (Squamata, Viperidae) on a swamp eel, genus *Synbranchus*. *Herpetology Notes* **11:**835–837.

**Emsley M. 1977.** Snakes, and Trinidad and Tobago. *Maryland Herpetological Society Bulletin* **13**:201–304.

**Farr WL, Lazcano D. 2017.** Distribution of *Bothrops asper* in Tamaulipas, Mexico and a review of prey items. *The Southwestern Naturalist* **62(1)**:77–84 DOI 10.1894/0038-4909-62.1.77.

**Fortier R. 2021.** *Bothrops asper* (Fer-de-Lance). Diet. *Herpetological Review* **52:**148.

**Fujishima K, Fukuyama R, Ishiba Y. 2021.** *Bothrops asper* (Fer-de-Lance). Diet and ophiophagy. *Herpetological Review* **52:**658.

**Gabrysova B, Aznar Gonzáles de Rueda J, Barrio-Amorós CL. 2020.** *Bothrops asper* (Terciopelo). Diet/Ophiophagy. *Herpetological Review* **51**:859–860.

**Greene HW. 1992.** The ecological and behavioral context for pitviper evolution. In: Greene HW, Campbell JA, Brodie ED, eds. *Biology of the Pitvipers.* Tyler, Texas: Selva Press, 107–117.

**Greene HW. 1997.** *Snakes: the evolution of mystery in nature.* Los Angeles, USA: University of California Press.

**Greene HW, Hardy DL. 1989.** Natural death associated with skeletal injury in the terciopelo, *Bothrops asper* (Viperidae). *Copeia* **1989(4)**:1036 DOI 10.2307/1445992.

**Hertz A, Natera M, Lotzkat S, Sunyer J, Mora D. 2009.** *Bothrops asper* (Mapanare, Lancehead). Prey. *Herpetological Review* **40**:230.

**Jones MA, Straka JR, Kayano K. 2014.** *Bothrops asper* (Fer-de-Lance). Diet. *Herpetological Review* **45**:3.

**Kuch U, Boada C, García F, Torres J, Freire A. 2004.** *Bothrops asper* (Terciopelo or equis). Diet. *Herpetological Review* **35**:273–274.

**Lemos-Espinal JA, Dixon JR. 2013.** *Amphibians and reptiles of San Luis Potosí.* Eagle Mountain, Utah: Eagle Mountain Publishing.

**Logan CJ, Montero C. 2009.** *Bothrops asper* (Terciopelo) scavenging behavior. *Herpetological Review* **40:**352–352 DOI 10.17863/CAM.5932.

**Londoño-Quiceno C, Escobar-Lasso S, Zuluaga-Isaza JC, Caicedo-Martínez LS. 2020.** Predation on Colombian endemic frog *Rheobates palmatus* (Werner, 1899) (Anura: Aromobatidae) by the Terciopelo viper *Bothrops asper* (Garman, 1884) (Squamata: Viperidae). *Herpetology Notes* **13:**641–644.

**Mata-Lorenzen J, Solórzano A. 2021.** *Bothrops asper* (Fer-de-lance) diet. *Herpetological Review* **52**:148–149.

**Mole RR. 1924.** The Trinidad snakes. *Proceedings of the Zoological Society of London* **94(1)**:235–278 DOI 10.1111/j.1096-3642.1924.tb01500.x.

**Moody EK. 2015.** *Bothrops asper* (Fer-de-Lance). Diet and feeding behavior. *Herpetological Review* **46:**266–267.

**Murphy JC. 1997.** *Amphibians and reptiles of Trinidad and Tobago.* Malabar, Florida: Krieger.

**Nicéforo-María H. 1930.** Los reptiles y batracios de Honda (Tolima) en el Museo de La Salle. *Revista Sociedad Colombiana de Ciencias Naturales* **19**:96–104.

**Parker HW. 1926.** The reptiles and batrachians of Gorgona Island, Colombia. *Annals and Magazine of Natural History* **17**(101):549–554 DOI 10.1080/00222932608633442.

**Picado C. 1931.** *Serpientes venenosas de Costa Rica.* San José, Costa Rica: Imprenta Alsina. Sauter, Arias & Co.

**Platt SG, Rainwater TR, Meerman JC, Miller SM. 2016.** Notes on the diet, foraging behavior, and venom of some snakes in Belize. *Mesoamerican Herpetology* **1**:162–170.

**Rojas Murcia LE, Carvajal Cogollo JE, Cabrejo Bello JA. 2016.** Reptiles from the seasonal dry forest the Caribbean region: distribution of habitat and use of food resource. *Acta Biologica Colombiana* **21:**365–377 DOI [10.15446/abc.v21n2.49393](https://doi.org/10.15446/abc.v21n2.49393).

**Roldan JSM, Lucero MF. 2011.** A prey item not previously recorded for *Bothrops asper*: a case of ophiophagy involving two sympatric pit viper species. *Herpetotropicos: Tropical Amphibians & Reptiles* **5(2)**:107–109.

**Ryan M, Blea N, Latella I, Kull M. 2010.** *Leptodactylus savagei* (smoky jungle frog). Antipredator defense. *Herpetological Review* **41**:337–338.

**Sasa M, Wasko DK, Lamar WW. 2009.** Natural history of the terciopelo *Bothrops asper* (Serpentes: Viperidae) in Costa Rica. *Toxicon* **54(7)**:904–922 DOI 10.1016/j.toxicon.2009.06.024.

**Segovia-Núñez G, de Osma A, Ramírez-Barajas P. 2014.** *Bothrops asper* (Terciopelo). Diet. *Herpetological Review* **45**:512–513.

**Sexton OJ, Heatwole H. 1965.** Life history notes on some Panamanian snakes*. Caribbean Journal of Science* **5:**39–43.

**Smith HM. 1947.** Notes on Mexican amphibians and reptiles. *Journal of the Washington Academy of Sciences* **37**:408–412.

**Solórzano A. 2004.** *Serpientes de Costa Rica: distribución, taxonomía e historia natural.* Costa Rica: Editorial INBio.

**Solórzano A, Cerdas L. 1989.** Reproductive biology and distribution of the terciopelo, *Bothrops asper* Garman (Serpentes: Viperidae), in Costa Rica. *Herpetologica* **45**:444–450.

**Sosa-Bartuano A, Añino Ramos Y, Santos A. 2017.** *Bothrops asper* (Garman, 1883). Diet. *Mesoamerican Herpetology* **4**:423–424.

**Stuart LC. 1948.** *The amphibians and reptiles of Alta Verapaz, Guatemala.* Ann Arbor: University of Michigan Press.

**Szczygieł H, Page R. 2020.** When the hunter becomes the hunted: foraging bat attacked by pit viper at frog chorus. *Ecology* **101(10)**:e03111 DOI 10.1002/ecy.3111.

**Urbina-Cardona JN. 2009.** *Bothrops asper* (Terciopelo). Diet. *Herpetological Review* **40:**94.

**Valencia JH, Garzón-Tello K, Barragán-Paladines ME, Oxford P. 2016.** *Serpientes venenosas del Ecuador: sistemática, taxonomía, historia natural, conservación, envenenamiento y aspectos antropológicos.* Quito, Ecuador: Fundación Herpetológica Gustavo Orcés.

**Vela RA, Juárez JLC, Calvario ÁIC. 2020.** Predation on Rainbow Ameivas, *Holcosus undulatus* (sensu lato), and a second record of predation on *H. amphigrammus* (Smith and Laufe 1945) by a terciopelo (*Bothrops asper*) in Veracruz, Mexico. *Reptiles & Amphibians* **27**:422–425 DOI 10.17161/randa.v27i3.14860.

**Voss RS. 2013.** Opossums (Mammalia: Didelphidae) in the diets of Neotropical pitvipers (Serpentes: Crotalinae): evidence for alternative coevolutionary outcomes? *Toxicon* **66(6)**:1–6 DOI 10.1016/j.toxicon.2013.01.013.
